# In vitro metabolism of Benzyl-4CN-BUTINACA and MDMB-4CN-BUTINACA using human hepatocytes and LC-QToF-MS analysis

**DOI:** 10.1007/s00204-025-04018-y

**Published:** 2025-03-18

**Authors:** Caitlyn Norman, Kristin Webling, Dārta Štālberga, Lisa Maas, Johannes Tveit, Huiling Liu, Shimpei Watanabe, Svante Vikingsson, Henrik Green

**Affiliations:** 1 Department of Biomedical and Clinical Sciences, Division of Clinical Chemistry and Pharmacology, Linköping University, Linköping, Sweden; 2 Chiron AS, Trondheim, Norway; 3 Department of Forensic Genetics and Forensic Toxicology, National Board of Forensic Medicine, Linköping University, Linköping, Sweden; 4 Forensic Science Group, Photon Science Research Division, RIKEN SPring-8 Center, Sayo, Hyogo Japan; 5 Center for Forensic Science Advancement and Application, RTI International, Research Triangle Park, NC USA

**Keywords:** New psychoactive substances, Synthetic cannabinoid receptor agonists, Metabolism, Cyanogenic, Human hepatocytes

## Abstract

Synthetic cannabinoid receptor agonists (SCRAs) are a large and continuously evolving group of new psychoactive substances (NPS). Recently, many different nitrile-containing SCRAs have emerged on the illicit market, two of which have been found to release cyanide during metabolism. This can produce symptoms similar to those of cyanide poisoning, contributing to the toxicity of these SCRAs. Notified by the EU Early Warning System in 2020, Benzyl-4CN-BUTINACA (Benzyl-4CN-BINACA, BZ-4CN-BUTINACA) is the most recent nitrile-containing SCRA to emerge. This study characterized the metabolism of Benzyl-4CN-BUTINACA and the prophetic compound MDMB-4CN-BUTINACA for the first time using ultra-high performance liquid chromatography coupled with quadrupole time-of-flight mass spectrometry (UHPLC-QToF-MS) following incubation with primary human hepatocytes (HHeps; 5 µmol/L, up to 5 h). For Benzyl-4CN-BUTINACA, nine metabolites (no phase II metabolites) were identified and 12 for MDMB-4CN-BUTINACA, including only two minor phase II metabolites. By far the most abundant metabolites for Benzyl-4CN-BUTINACA were metabolites with a dihydrodiol on the indazole core (B1) and decyanation to a carboxylic acid (B2). The metabolites with ester hydrolysis (M1) and ester hydrolysis with dehydrogenation (M2) were the most abundant for MDMB-4CN-BUTINACA. Decyanation was less prevalent for these compounds than for other nitrile-containing SCRAs, such as Cumyl-4CN-BUTINACA, with only 29.0% and 1.78% of metabolites of Benzyl-4CN-BUTINACA and MDMB-4CN-BUTINACA, respectively, having a loss of cyanide. However, the second major metabolite of Benzyl-4CN-BUTINACA was a decyanation metabolite, making the potential CN formation not negligible.

**Supplementary Information:**

The online version contains supplementary material available at 10.1007/s00204-025-04018-y.

## Introduction

By the end of 2023, the European Union Drugs Agency (EUDA), formerly the European Monitoring Centre for Drugs and Drug Addiction (EMCDDA), were monitoring over 950 new psychoactive substances (NPS), where synthetic cannabinoid receptor agonists (SCRAs) are one of the fastest growing groups (EMCDDA 2024). SCRAs are designed to activate the cannabinoid receptors, CB_1_ and CB_2_, and mimic the effects of Δ^9^-tetrahydrocannabinol, Δ^9^-THC, the major psychoactive component of cannabis (EMCDDA 2009); however, Δ^9^-THC is a partial agonist of the CB receptors, whereas SCRAs are typically full agonists. The often significantly greater potency of SCRAs leads to more severe effects (Castaneto et al. 2014) that more closely resemble the adverse effects of psychostimulants than Δ^9^-THC (Giorgetti et al. 2020; Darke et al. 2021). In addition, SCRAs are rapidly and extensively metabolized, making the detection of the use of SCRAs through the analysis of biological fluids more challenging (Watanabe et al. 2017a).

Several approaches are available to determine SCRA metabolism, including in vitro*, *in silico*, *in vivo, in zebrafish larvae, and using the fungus *Cunninghamella elegans* (Diao and Huestis 2019). The most commonly used methods are in vitro using human liver microsomes (HLMs) (Kim et al. 2016; Öztürk et al. 2018; Yeter and Ozturk 2019) or human hepatocytes (HHeps) (Castaneto et al. 2015; Diao et al. 2017; Åstrand et al. 2018; Watanabe et al. 2020a). HLM incubation is the most popular in vitro metabolism model due to its affordability and simplicity in comparison to HHeps. However, HLM incubation does not produce any phase II metabolites, so HHeps incubation, which uses isolated living cells, often provides a better representation of liver metabolism. These methods are often used together and ideally, compared to the metabolic results from the analysis of genuine urine and blood samples. Research into the metabolism of SCRAs is required to support forensic and clinical toxicologists in their detection of SCRA use in biological fluids especially for newly emerged SCRAs whose metabolites are typically unknown (Diao and Huestis 2019).

**Fig. 1:**
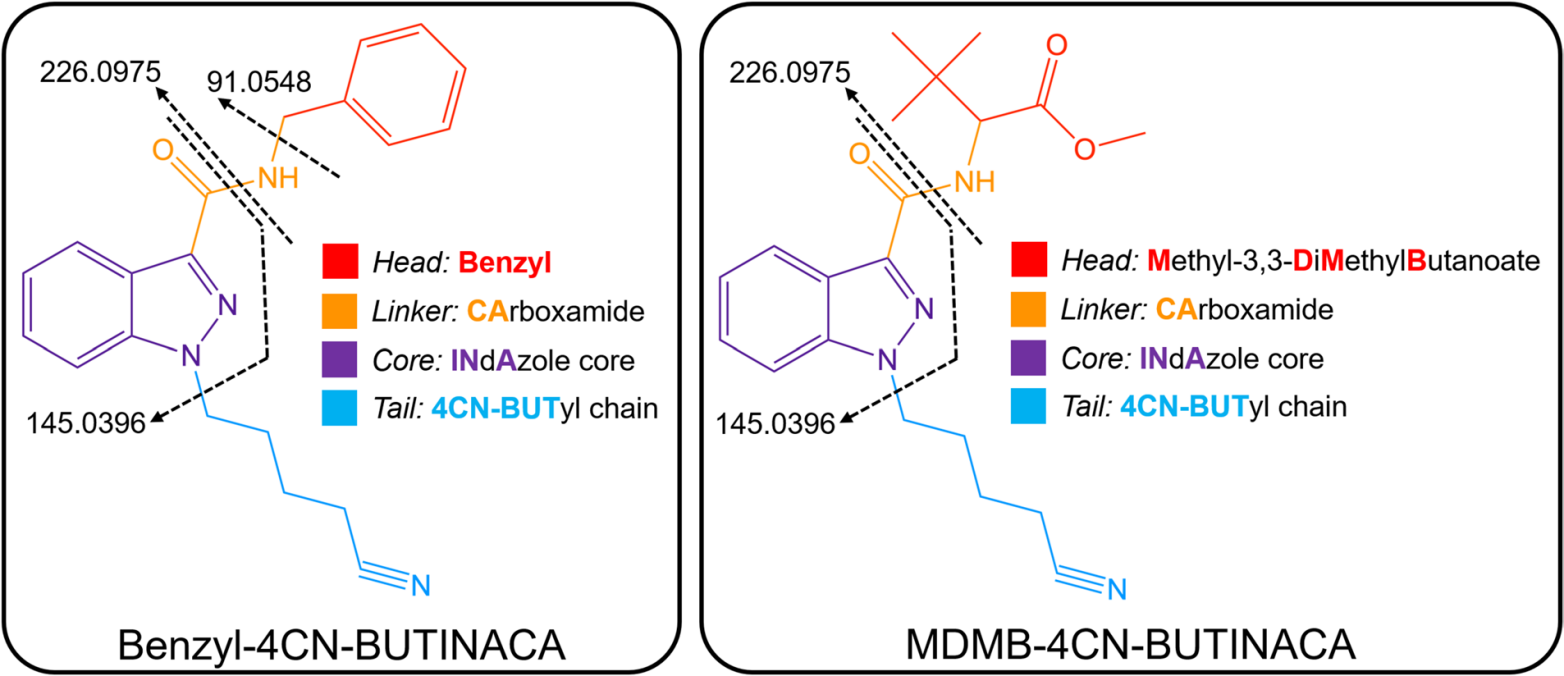
Structures of SCRAs examined in this study, Benzyl-4CN-BUTINACA and MDMB-4CN-BUTINACA, where the four structural moieties are indicated by color and the fragmentation patterns are indicated in black with dashed arrows (color figure online)

The SCRAs available on the illicit market are continuously evolving, often in response to national and international legislation (Reuter and Pardo 2017; Norman et al. 2021). SCRAs are often characterized based on four main structural components (see Fig. 1): the head (also known as linked group), linker, core, and tail. The tail, linker, and head groups have been found to affect the binding in the CB receptors, whereas the core and head groups affect the potency (Krishna Kumar et al. 2019). Many of the most potent and prevalent SCRAs on the illicit drug market have been indole- or indazole-3-carboxamide SCRAs (ICA or INACA, respectively) with variation seen in the head and tail groups (Banister and Connor 2018; Wouters et al. 2019; Norman et al. 2020). Valine (MMB, AMB), *tert-*leucine (MDMB), valinamide (AB), and leucinamide (ADB, ADMB) are the most common head groups as they typically result in the greatest potency, but many other head groups have been detected, including adamantyl (A), cumyl, and benzyl (BZ). The tail group is often a butyl or pentyl alkyl chain but with different substitutions often emerging, such as the addition of a fluorine, alkene, or nitrile (Banister and Connor 2018; Patel et al. 2021).

Many different nitrile-containing SCRAs have recently emerged on the illicit market, including AB-4CN-BUTICA (AB-4CN-BICA) (US Drug Enforcement Administration Diversion Control Division 2021; Sparkes et al. 2022), MMB-4CN-BUTINACA (MMB-4CN-BINACA, AMB-4CN-BUTINACA, AMB-4CN-BINACA) (Sparkes et al. 2022), and Cumyl-4CN-BUTINACA (Cumyl-4CN-BINACA) (EMCDDA 2017; Åstrand et al. 2018; Expert Committee on Drug Dependence 2018; Öztürk et al. 2018; Kevin et al. 2019; Norman et al. 2020). Benzyl-4CN-BUTINACA (Benzyl-4CN-BINACA, BZ-4CN-BUTINACA) is the most recent nitrile-containing SCRA to emerge and was first notified by the EMCDDA through the EU Early Warning System in March 2020 following its detection in herbal material seized by Swedish Customs in November 2019 (EMCDDA 2020). The metabolism of this new SCRA has not previously been examined, but many drugs and pharmaceuticals containing an aliphatic nitrile, including Cumyl-4CN-BUTINACA, have been found to release cyanide in vivo from the metabolized nitrile. It has been suggested that this release of cyanide contributes to the toxicity of Cumyl-4CN-BUTINACA (Grogan et al. 1992; Åstrand et al. 2018; Staeheli et al. 2018; Kevin et al. 2019).

This study aimed to characterize the metabolism of Benzyl-4CN-BUTINACA and the prophetic compound MDMB-4CN-BUTINACA and determine the extent of decyanation (i.e., cyanide release) using ultra-high performance liquid chromatography coupled with quadrupole time-of-flight mass spectrometry (UHPLC-QToF-MS) following incubation with primary HHeps.

## Materials and methods

### Chemicals and reagents

Cryopreserved HHeps (LiverPool, 10 donor pool) and inVitro Gro HT thawing medium were obtained from Bioreclamation IVT (Brussels, Belgium). LC–MS grade acetonitrile, methanol, water, and formic acid; Williams E medium; HEPES buffer; L-glutamine; trypan blue 0.4% solution; the internal standard solution mixture of 15 μg/mL D_8_-amphetamine, 5 μg/mL D_5_-diazepam, 2.5 μg/mL D_3_-mianserin, and 15 μg/mL D_5_-phenobarbital, and the positive control solution of 5 mg/mL caffeine, 1 mg/mL bupropion, 1 mg/mL diclofenac, 1 mg/mL omeprazole, 1 mg/mL dextromethorphan, 1 mg/mL chlorzoxazone, and 1 mg/mL midazolam were obtained from Thermo Fisher Scientific (Gothenburg, Sweden). Sample preparation used high-purity water made on site using a MilliQ Gradient production unit from Millipore (Billerica, MA, USA).

The Benzyl-4CN-BUTINACA (95.4% chromatographic purity) and MDMB-4CN-BUTINACA (99.9% chromatographic purity) reference standards used in this study were synthesized as part of the EU EUREKA funded NPS-REFORM project, a collaboration between Chiron AS (Trondheim, Norway) and Linköping University.

### Hepatocyte incubation

The HHep incubations were performed according to the previously described protocol by Watanabe et al. (Watanabe et al. 2017b). In short, the cryopreserved HHeps were thawed and transferred to 48 mL inVitro Gro HT media preheated to 37 °C, centrifuged at room temperature for 5 min at 100×*g*, and washed twice with 50 mL Williams E medium supplemented with 2 mM L-glutamine and 20 mM HEPES buffer. The cell concentration was adjusted to 2 × 10^6^ cells/mL with supplemented Williams E medium using the 0.4% trypan blue exclusion method.

The 1 mg/mL SCRA solutions were diluted to 10 μM in supplemented Williams E medium. 50 μL of this SCRA working solution and 50 μL of the HHep solution were mixed in 96-well plates with a final SCRA solution of 5 μmol/L. The HHeps were incubated at 37 °C for 0, 0.5, 1, and 3 h for Benzyl-4CN-BUTINACA and 0, 1, 3, and 5 h for MDMB-4CN-BUTINACA. The reactions were quenched by 100 μL ice-cold acetonitrile mixed with the internal standard solution (diluted in methanol with final concentrations of 150 ng/mL D_8_-amphetamine, 50 ng/mL D_5_-diazepam, 25 ng/mL D_3_ mianserin, and 150 ng/mL D_5_-phenobarbital), except for the 0 h, in which ice-cold acetonitrile was added prior to the addition of the HHeps. The control samples were incubated for 3 h where the positive control consisted of HHeps and the positive control stock (diluted in methanol with the final concentration of 500 μM), which contains compounds selectively metabolized by CYP 450 1A2, 2B6, 2C9, 2C19, 2D9, 2E1, and 3A4; the negative control consisted of HHeps and medium; and the degradation control consisted of SCRA solutions and medium. After terminating the reaction, the plates were centrifuged at 1100 × g at 4 °C for 15 min to ensure that no cells or cell debris, i.e., large proteins, could negatively affect the LC columns. 100 µL aliquots of supernatants were transferred to an injection plate, sealed, and stored at −20 °C until LC-QToF-MS analysis.

### LC–QToF–MS analysis

For the LC–QToF–MS analysis, 5 µL of the supernatant from the HHep and drug incubations were injected on an Agilent 1290 Infinity UHPLC system (Agilent Technologies, Kista, Sweden) with an Acquity HSS T3 column (150 × 2.1 mm, 1.8 μm; Waters, Sollentuna, Sweden) fitted with an Acquity VanGuard precolumn (Waters) coupled with an Agilent 6550 iFunnel QToF-MS (Agilent Technologies) with a dual Agilent Jet Stream electrospray ionization source. Mobile phases (A) 0.1% formic acid in water and (B) 0.1% formic acid in acetonitrile were used in gradient mode: 1% B (0–0.6 min); 1–20% B (0.6–0.7 min); 20–85% B (0.7–13 min); 85–95% B (13–15 min); 95% B (15–18 min); 95–1% B (18–18.1 min); 1% B (18.1–19 min). The flow rate was 0.5 mL/min and the column temperature was 60 °C.

MS data were acquired in positive electrospray ionization mode using Auto MS/MS acquisition with the following parameters: scan range, 100–950 m/z (MS) and 50–950 m/z (MS/MS); precursor intensity threshold, 5000 counts; precursor number per cycle, 5; fragmentor voltage, 380 V; collision energy, 3 eV at 0 m/z ramped up by 8 eV per 100 m/z; gas temperature, 150ºC; gas flow, 18 L/min; nebulizer gas pressure, 345 kPa; sheath gas temperature, 375 °C; and sheath gas flow, 11 L/min.

### Data analysis

The results obtained from the LC–QToF–MS were analyzed using the Agilent MassHunter Qualitative Analysis software (version B.07.00). A library of potential metabolites was created based on biotransformations of similar compounds reported in the literature. A search of the library was performed with the following parameters: mass error, 30 ppm (this was set higher to account for the potentially high mass errors from saturated peaks); absolute peak area threshold, > 20,000 counts; maximum number of matches, 10; and extraction window, 100 ppm. The metabolites were identified if mass errors of protonated metabolites were < 5 ppm (unless saturated peak), the retention time was plausible (between 4 and 15 min), and the peak was absent in negative controls and degradation controls. All the biotransformations and potential structures of the metabolites were identified based on the HRMS m/z and the MS/MS spectra. Further confirmation was based on the retrieved MS/MS fragmentation pattern, where the MS/MS spectrum had to be available in at least two samples for the metabolite to be confirmed.

## Results

The fragmentation pattern of Benzyl-4CN-BUTINACA and MDMB-4CN-BUTINACA is shown in Fig. 1. In QToF-MS analysis, Benzyl-4CN-BUTINACA (m/z 333.1710) was fragmented into four major product ions: m/z 91.0548, representing the benzene ring and methyl group from the head moiety; m/z 145.0396, representing the indazole acylium ion; m/z 226.0975, representing the indazole acylium ion and 4CN-butyl tail moiety; and m/z 316.1444, representing the parent structure without the ammonium ion. The observed fragment ions were used as the basis for elucidating the structures of the metabolites.

MDMB-4CN-BUTINACA (m/z 371.2078) was fragmented into two major product ions: m/z 145.0396, representing the indazole acylium ion, and m/z 226.0975, representing the indazole acylium ion and 4CN-butyl tail moiety. The observed fragment ions were used as the basis for elucidating the structures of the metabolites.

### Benzyl-4CN-BUTINACA

**Fig. 2:**
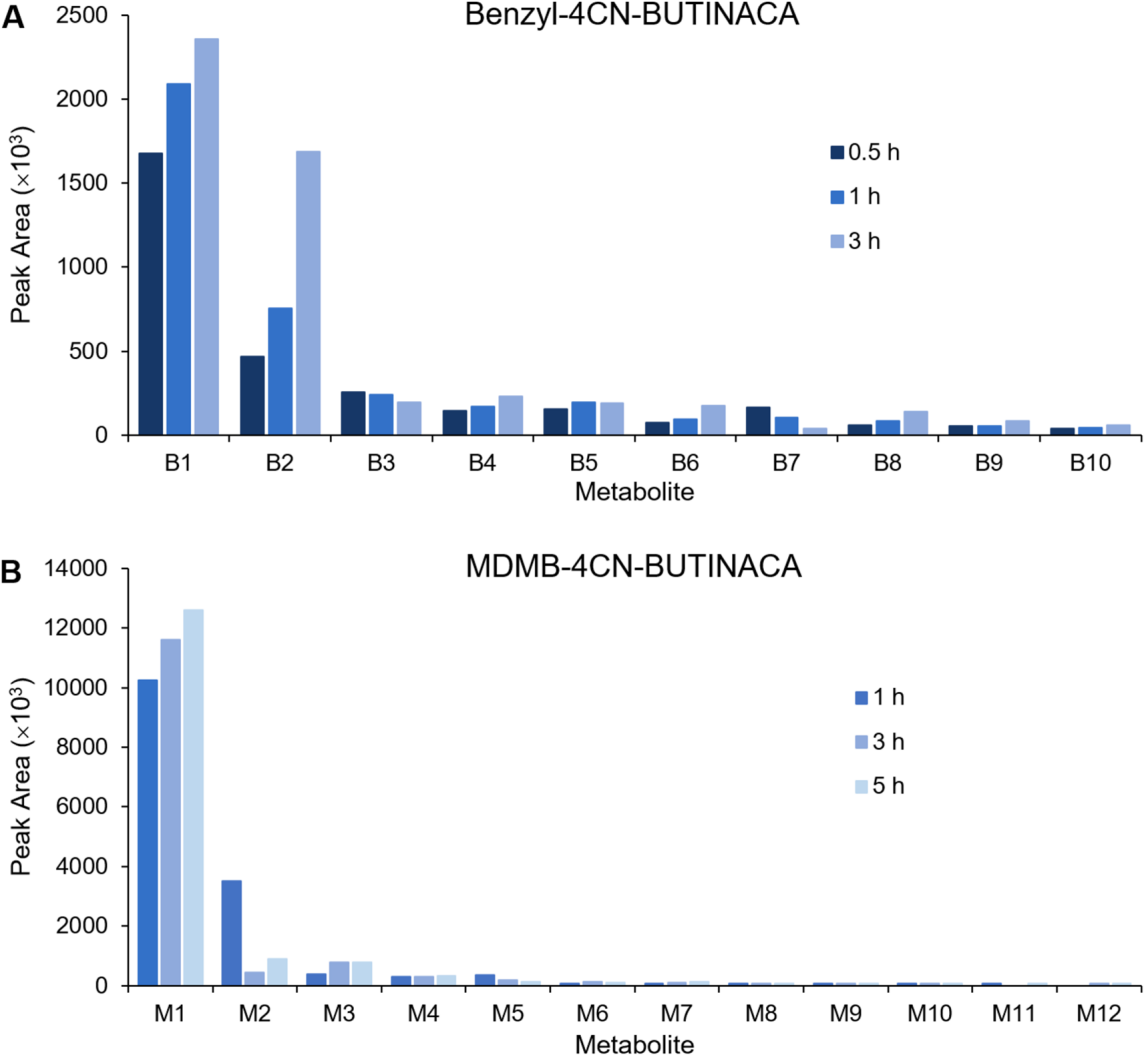
Average peak areas of the metabolites of **A** Benzyl-4CN-BUTINACA after 0.5-, 1-, and 3-h incubations and **B** MDMB-4CN-BUTINACA after 1-, 3-, and 5-h incubations with HHeps for two replicate samples (averaged)

**Fig. 3:**
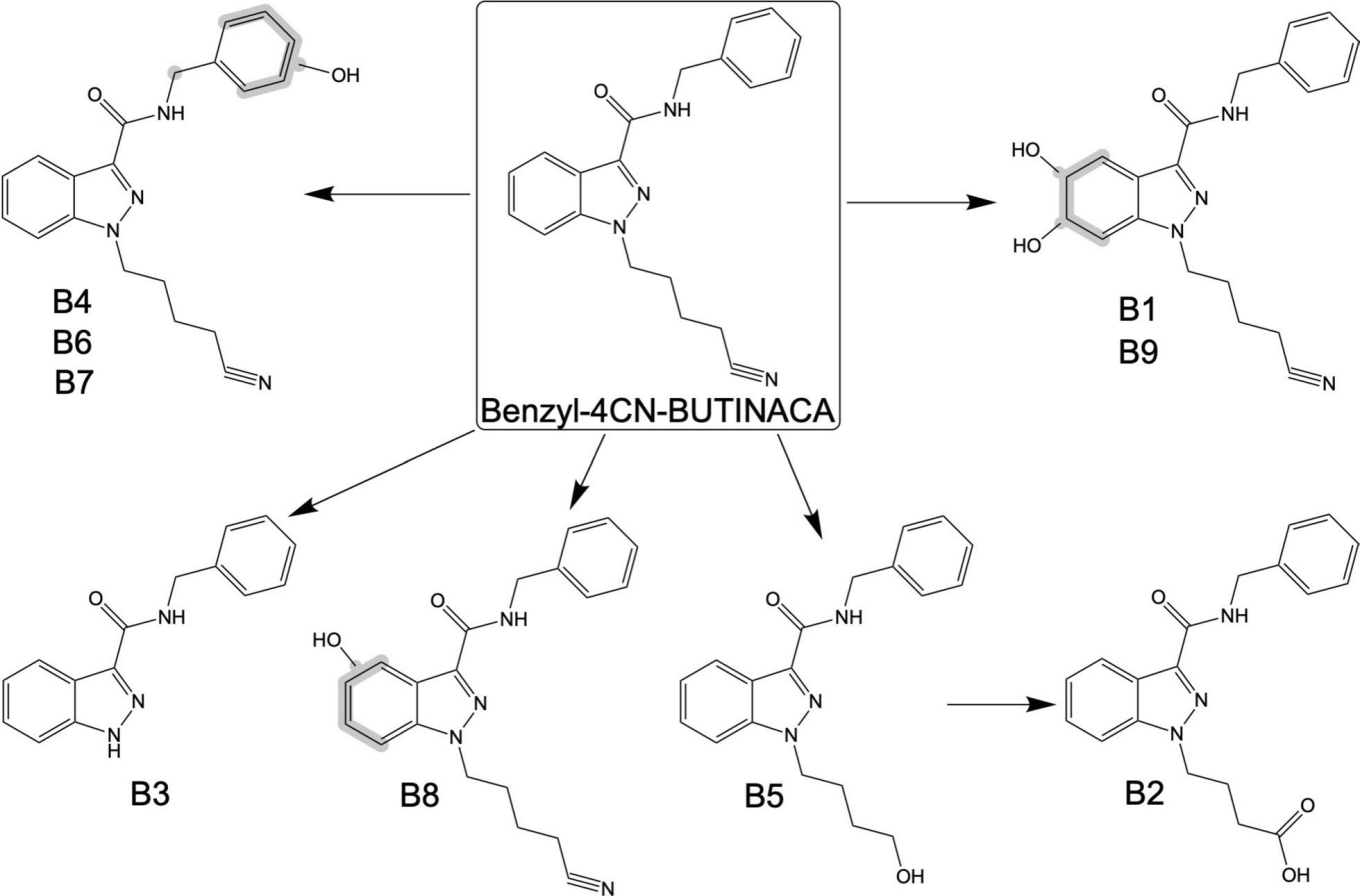
Proposed metabolic pathways of Benzyl-4CN-BUTINACA following duplicate 0.5-, 1-, and 3-h incubations with HHeps

Following incubation with HHeps, nine metabolites (B1-B9) were identified for Benzyl-4CN-BUTINACA. The metabolites eluted between 5.04 and 8.34 min with the parent drug eluting at 10.23 min (see Table 1). The observed biotransformations included monohydoxylations (MonoOH), dihydrodiol formation, *N*-dealkylation, and decyanation. There was no glucuronidation or other phase II biotransformation observed. Based on the total peak area of metabolites, most biotransformations occurred on the indazole core (55.1%) and butyl tail (34.8%), with only 10.1% of the metabolites having a biotransformation on the benzyl head. The identified metabolites are listed in Table 1 with the major fragment ions; respective retention times; molecular formulas; mass errors; peak areas of two replicates obtained from 0.5-, 1-, and 3-h incubations; and the percentage of the total peak area of metabolites across all incubations. The metabolites are numbered according to their total peak area, from the highest to the lowest area. The detection of all metabolites was reproducible, with all but B9 being observed across all time course samples across the HHep incubations (duplicate samples taken at three time points). The average peak areas of the two replicates obtained from each incubation for the metabolites are also illustrated in Fig. 2A. The proposed structures of the metabolites are organized in suggested metabolic pathways in Fig. 3. The mass spectra and proposed fragmentation patterns of the parent compound and all metabolites can be found in the Supplementary Information.

**Table 1:** Benzyl-4CN-BUTINACA metabolites with biotransformation; molecular formulas; mean retention times; exact (calculated) masses of the protonated molecules; mass errors from all samples; peak areas after 0.5-, 1-, and 3-h incubations for two replicate samples; the percentage of the total peak area of metabolites across all incubations; and major fragment ions (also indicative of biotransformation)

Met #	Biotransformation	Formula	Mean RT (min)	Exact mass [M + H]^+^ (m/z)	Average mass error (ppm)	#1 peak area ( × 10^3^)			#2 peak area ( × 10^3^)			%	Major fragment ions
						0.5 h	1 h	3 h	0.5 h	1 h	3 h		
	Benzyl-4CN-BUTINACA	C_20_H_20_N_4_O	10.23	333.1710	8.98	20,326	16,780	11,689	20,683	16,903	11,059	–	91.0548, 145.0396, 226.0975, 316.1444
B1	Dihydrodiol (indazole)	C_20_H_22_N_4_O_3_	5.39	367.1765	2.25	1731	2072	2399	1622	2114	2316	51.3	91.0548, 161.0346, 242.0930, 260.1035
B2	Decyanation to carboxylic acid	C_19_H_19_N_3_O_3_	8.20	338.1499	1.91	463	694	1608	477	819	1767	24.4	69.0335, 87.0441, 91.0548, 145.0396, 213.0659, 231.0770
B3	N-dealkylation	C_15_H_13_N_3_O	7.57	252.1131	1.78	250	219	186	260	260	206	5.8	91.0548, 145.0396
B4	MonoOH (head)	C_20_H_20_N_4_O_2_	5.35	349.1659	0.73	148	161	227	147	182	239	4.6	55.0542, 89.0386, 107.0497, 145.0396, 226.0975, 332.1394
B5	Decyanation to alcohol	C_19_H_21_N_3_O_2_	8.34	324.1707	0.93	157	174	180	158	219	206	4.6	91.0548, 131.0604, 145.0396, 199.0866, 217.0977
B6	MonoOH (head)	C_20_H_20_N_4_O_2_	5.04	349.1659	0.70	76	95	172	72	100	179	2.9	55.0542, 57.0669, 89.0386, 107.0497, 145.0396, 226.0975
B7	MonoOH (head)	C_20_H_20_N_4_O_2_	7.46	349.1659	0.46	149	99	39	180	111	44	2.6	89.0386, 107.0497, 145.0396, 226.0975
B8	MonoOH (indazole)	C_20_H_20_N_4_O_2_	6.06	349.1659	0.36	62	85	143	62	88	135	2.4	91.0548, 161.0346, 242.0930
B9	Dihydrodiol (indazole)	C_20_H_22_N_4_O_3_	6.06	367.1765	−0.42	54	51	87	n.d	57	81	1.4	55.0542, 91.0548, 133.0396, 161.0346, 242.0930

Metabolites are ordered from most to least abundant by average peak area across all incubations

*n.d.* not detected

The most abundant metabolite was the metabolite with a dihydrodiol (B1). These metabolites were characterized by the addition of m/z 34 to the mass of the parent, which is consistent with the addition of two oxygens and two hydrogens. The presence of the fragment ions at m/z 242.0930 and 260.1035, which indicate an addition of m/z 17 and 34, respectively, is consistent with the addition of a dihydrodiol to the indazole core followed by water losses. This was also supported by the presence of the fragment ion at m/z 161.0346, which indicates an addition of m/z 17, which is consistent with the addition of a dihydrodiol to the indazole core followed by a water loss. No modification to the fragment ion at m/z 91.0548 demonstrates no biotransformations on the benzyl head moiety. There was also a second metabolite with a dihydrodiol (B9) with a much smaller peak area for all incubations. The fragment ions of B9 were similar to B1, except B9 did not have fragment ions at m/z 226.0975 or 260.1035 and an additional fragment ion at m/z 133.0396, which represents the indazole acylium ion with a hydroxy group. Unfortunately, the exact locations of the dihydrodiols could not be determined for B1 and B9.

The second most abundant metabolite was the metabolite with decyanation to a carboxylic acid (B2), which results in the production of cyanide. The fragment ion at m/z 231.0770 corresponds to the indazole acylium ion with a butanoic acid chain and subsequent water loss generates m/z 213.0659. No modifications to the fragment ions at m/z 91.0548 and 145.0396 indicate no biotransformations on the benzyl head moiety or indazole core, respectively. The metabolite with decyanation to an alcohol (B5) is likely an intermediate to the metabolite with decyanation to a carboxylic acid (B2). Based on total peak area, decyanation consisted of 29% of metabolites.

The rest of the metabolites were observed with much smaller peak areas than metabolites B1 and B2 for all incubations (see Fig. 2A), making up only 24.3% of the total peak area across all incubations. MonoOH was the most common biotransformation observed (B4, B6–B8), where they most often occurred at the head moiety (B4, B6, and B7) but also the indazole core (B8). Unfortunately, the exact locations of the added hydroxy groups were unable to be determined for these metabolites. It should be noted that B8 may be in-source fragmentation of B9 through water loss (-m/z 18), but this could not be determined conclusively. The other dihydrodiol metabolite (B1) does not show in-source fragmentation, which would seem to support that B8 is not in-source fragmentation of B9. However, the in-source fragmentation of B1, which would be a metabolite with a monoOH at the indazole core (m/z 349.1659), could be hidden by the larger peak of B4, a metabolite with a monoOH on the head moiety (m/z 349.1659) that elutes at a similar retention time (5.35 min for B4 and 5.39 min for B1). Finally, there was also an *N*-dealkylation metabolite (B3), which only had fragment ions at m/z 91.0548 and 145.0396 indicating the lack of a butyl tail.

### MDMB-4CN-BUTINACA

**Fig. 4:**
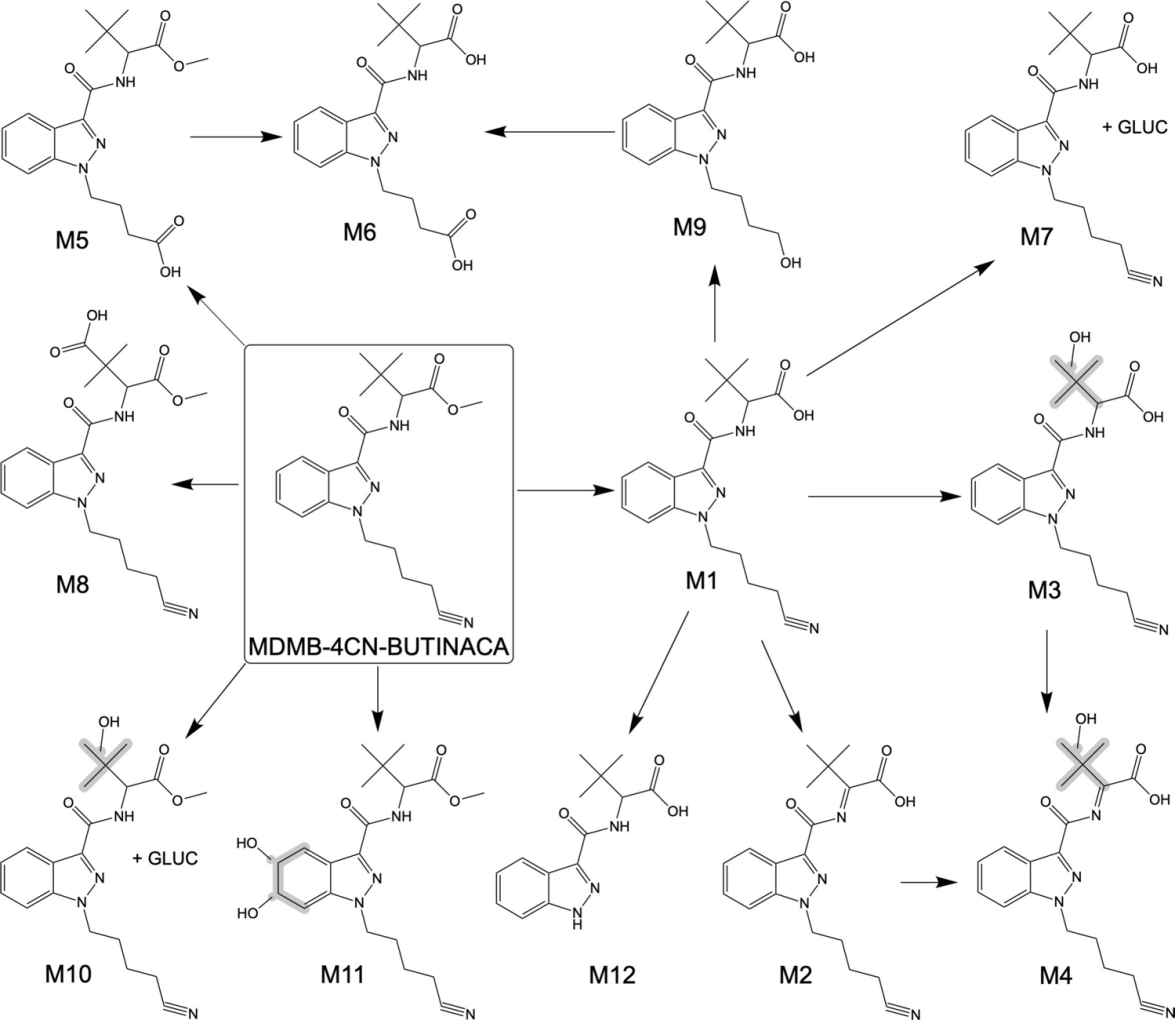
Proposed metabolic pathways of MDMB-4CN-BUTINACA following duplicate 1-, 3-, and 5-h incubation with HHeps

Following incubation with HHeps, 12 metabolites (M1-M12) were identified for MDMB-4CN-BUTINACA. The metabolites eluted between 5.23 and 7.78 min with the parent drug eluting at 9.33 min (see Table 2). It should be noted that the mass errors for MDMB-4CN-BUTINACA and M1 were high (28.46 and 10.40 ppm, respectively); however, this was considered acceptable as the shapes of the chromatographic peaks indicated saturation of the detector, which is known to lead to large mass errors. The observed biotransformations included ester hydrolysis, monoOH, dehydration (dehyd), carboxylation, dihydrodiol formation, *N*-dealkylation, decyanation, and glucuronidation. Only two metabolites (M7 and M10) were glucuronidated with no other phase II metabolites observed. The vast majority of biotransformations occurred at the head moiety (98.3% of total peak area of metabolites), with only 2.5 and 0.1% of the total peak area of metabolites having a biotransformation on the butyl tail and indazole core, respectively. The identified metabolites are listed in Table 2 with the diagnostic fragment ions; respective retention times; molecular formulas; mass errors; peak areas of two replicates obtained from 1-, 3-, and 5-h incubations; and the percentage of the total peak area of metabolites across all incubations. The metabolites are numbered according to their total peak area, from the highest to the lowest area. The detection of all metabolites was reproducible, with all but two (M11 and M12) being observed across all time course samples across the HHep incubations (duplicate samples taken at three time points). The average peak areas of the two replicates obtained from each incubation for the metabolites are also illustrated in Fig. 2B. The proposed structures of the metabolites are organized in suggested metabolic pathways in Fig. 4. The mass spectra and proposed fragmentation patterns of the parent compound and all metabolites can be found in the Supplementary Information.

**Table 2:** MDMB-4CN-BUTINACA metabolites with biotransformation; molecular formulas; mean retention times; exact (calculated) masses of the protonated molecules; mass errors from all samples; peak areas after 1-, 3-, and 5-h incubations for two replicate samples; the percentage of the total peak area of metabolites across all incubations; and major fragment ions (also indicative of biotransformation)

Met #	Biotransformation	Formula	Mean RT (min)	Exact mass [M + H]^+^ (m/z)	Average mass error (ppm)	#1 peak area ( × 10^3^)			#2 peak area ( × 10^3^)			%	Major fragment ions
						1 h	3 h	5 h	1 h	3 h	5 h		
	MDMB-4CN-BUTINACA	C_20_H_26_N_4_O_3_	9.33	371.2078	28.46	14,300	251	936	15,200	284	934	–	145.0396, 226.0975
M1	Ester hydrolysis	C_19_H_24_N_4_O_3_	7.42	357.1921	10.40	10,200	11,400	12,400	10,300	11,800	12,800	78.6	145.0396, 226.0975
M2	Ester hydrolysis + dehyd	C_19_H_22_N_4_O_3_	7.21	355.1765	2.30	3440	429	799	3590	489	987	11.1	145.0396, 226.0975
M3	Ester hydrolysis + monoOH (*tert*-butyl)	C_19_H_24_N_4_O_4_	5.49	373.1870	1.03	383	775	733	380	804	829	4.5	145.0396, 226.0975
M4	Ester hydrolysis + dehyd + monoOH (*tert*-butyl)	C_19_H_22_N_4_O_4_	6.26	371.1714	−0.77	296	306	304	302	316	345	2.1	145.0396, 226.0975
M5	Decyanation to carboxylic acid	C_19_H_25_N_3_O_5_	7.78	376.1867	−0.17	356	186	100	363	212	157	1.6	87.0441, 145.0396, 231.0764
M6	Ester hydrolysis + decyanation to carboxylic acid	C_18_H_23_N_3_O_5_	6.12	362.1710	−1.62	60	133	102	59	142	119	0.7	87.0441, 145.0396, 231.0764
M7	Ester hydrolysis + GLUC	C_25_H_32_N_4_O_9_	5.86	533.2242	−2.13	45	94	126	42	96	130	0.6	145.0397, 226.0971
M8	Carboxylation (*tert*-butyl)	C_20_H_24_N_4_O_5_	6.67	401.1819	−1.36	38	45	47	36	44	44	0.3	55.0542, 145.0396, 226.0975
M9	Ester hydrolysis + decyanation to alcohol	C_18_H_25_N_3_O_4_	6.21	348.1918	−3.24	21	38	29	21	42	35	0.2	73.0648, 86.0964, 145.0396, 217.0972
M10	MonoOH (*tert*-butyl) + GLUC	C_26_H_34_N_4_O_10_	5.23	563.2348	−1.59	27	32	29	30	33	25	0.2	145.0396, 226.0975
M11	Dihydrodiol (indazole)	C_20_H_28_N_4_O_5_	5.73	405.2132	−2.92	31	n.d	n.d	31	n.d	23	0.1	161.0346, 242.0924, 260.1030
M12	*N*-dealkylation + ester hydrolysis	C_14_H_17_N_3_O_3_	5.71	276.1343	−2.94	n.d	n.d	22	n.d	22	n.d	0.1	86.0964, 145.0396

Metabolites are ordered from most to least abundant by average peak area across all incubations

*n.d.* not detected

The metabolite with ester hydrolysis (M1) was by far the most abundant metabolite with about a three times greater peak area than the next most abundant metabolite in the 1 h samples and about ten times greater in the 3 and 5 h samples (see Fig. 2B). This metabolite was characterized by the reduction of m/z 14, which is consistent with a replacement of the ester on the *tert*-leucine head moiety with a carboxylic acid group, with the same two major fragment ions observed as for the parent, indicating no further modifications.

The rest of the metabolites were observed with much smaller peak areas than metabolite M1 for all incubations (see Fig. 2B), making up only 21.4% of the total peak area across all incubations. Ester hydrolysis of the *tert*-leucine head moiety was the most common biotransformation observed (M1–M4, M6–M7, M9, and M12), where all metabolites had undergone ester hydrolysis apart from the metabolite with decyanation to carboxylic acid (M5), metabolite with carboxylation on the *tert*-butyl section of the head moiety (M8), metabolite with monoOH on the *tert*-leucine head moiety and glucuronidation (M10), and metabolite with dihydrodiol formation of the indazole core moiety (M11). Based on total peak area, ester hydrolysis occurred in 97.8% of all metabolites.

Decyanation and monoOH were the second most common biotransformation, being observed in three metabolites (M5, M6, and M9 and M3, M4, and M10, respectively). The metabolite with decyanation to a carboxylic acid (M5) was formed directly from the parent, whereas the metabolite with decyanation to a carboxylic acid and ester hydrolysis (M6) is likely derived from either M5 or the metabolite with decyanation to an alcohol and ester hydrolysis (M9) as an intermediate. Only 1.78% of MDMB-4CN-BUTINACA metabolites had decyanation. The presence of the m/z 226.0975 fragment for all three of the metabolites with monoOH in combination with other biotransformations (M3, M4, and M10) suggests the *tert*-butyl chain as the site of hydroxylation.

## Discussion

For Benzyl-4CN-BUTINACA, a total of nine metabolites were identified following incubation with HHeps. The two most abundant metabolites were a dihydrodiol (B1) and a metabolite with decyanation to a carboxylic acid (B2), which comprised 74.8% of the total peak area across all incubations. There was an additional metabolite with a dihydrodiol (B9), although it was much less abundant. For SCRAs, it has been suggested that the dihydrodiol is formed on the indole/indazole core via epoxide formation followed by epoxide hydrolysis (Wintermeyer et al. 2010; Kim et al. 2016; Watanabe et al. 2020a, b). Within the literature, the metabolite with a dihydrodiol on the indazole core was reported to be the most abundant metabolite for ADB-BUTINACA following incubation with HHeps (Kronstrand et al. 2022). It was also reported as a metabolite for other indole- and indazole-containing SCRAs [e.g., JWH-200 (De Brabanter et al. 2013), ADB-FUBINACA (Carlier et al. 2017), AB-FUBINACA (Castaneto et al. 2015), MDMB-5’Br-BUTINACA (Norman et al. 2024)], but as a relatively minor metabolite. This was also the case for MDMB-4CN-BUTINACA in this study, where the dihydrodiol metabolite consisted of less than 1% of the total peak area across all incubations.

As shown in Fig. 2A, the eight remaining metabolites of Benzyl-4CN-BUTINACA comprised only 25.2% of the total peak area across all incubations. Based on the total peak area of metabolites, the indazole core and butyl tail were the main sites of biotransformations for Benzyl-4CN-BUTINACA with only 10% of biotransformations occurring on the benzyl head moiety. This is similar to the metabolism from HHeps incubation of another SCRA with a benzyl head moiety, SDB-006 (Benzyl-PICA), where only 8% of the total peak area of the metabolites had a biotransformation on the benzyl head moiety. However, debenzylation was the only biotransformation on the benzyl head moiety found for SDB-006 (Diao et al. 2017), whereas no debenzylation was observed in this study for Benzyl-4CN-BUTINACA.

A total of 12 metabolites were identified for MDMB-4CN-BUTINACA following incubation with HHeps, where the most abundant metabolite by far had an ester hydrolysis (M1). Ester hydrolysis was the main biotransformation for MDMB-4CN-BUTINACA, occurring in 97.8% of all metabolites. The remaining metabolites of MDMB-4CN-BUTINACA comprised only 21.4% of the total peak area across all incubations (Fig. 2B), where 98.3% of all biotransformations occurred on the head moiety based on the total peak area of metabolites. These results are consistent with the metabolism of other SCRAs with a *tert*-leucine head moiety [e.g., 4F-MDMB-BUTINACA (Haschimi et al. 2019; Leong et al. 2021), 5F-MDMB-PINACA (Kusano et al. 2018; Yeter and Ozturk 2019), MDMB-4en-PINACA (Erol Ozturk and Yeter 2020; Watanabe et al. 2020a)], where a metabolite with ester hydrolysis was the most abundant metabolite and ester hydrolysis was the most prevalent biotransformation.

There were no phase II biotransformations observed for Benzyl-4CN-BUTINACA and only two metabolites with the phase II biotransformation of glucuronidation observed for MDMB-4CN-BUTINACA, although it only accounted for 0.8% of the total peak area of the metabolites. The little to no glucuronidated metabolites for these SCRAs indicates hydrolysis of urine samples may not be necessary to identify their use; however, SCRA metabolites may be more extensively glucuronidated in urine samples than following HHeps incubation. For example, for Cumyl-4CN-BUTINACA, Åstrand et al. found only 1.9% of metabolites from HHeps incubation to be glucuronidated versus 28.3% of metabolites from urine samples (Åstrand et al. 2018).

29% of Benzyl-4CN-BUTINACA metabolites had a loss of cyanide, while only 1.78% of MDMB-4CN-BUTINACA metabolites had a loss of cyanide. However, it should be noted that there were only two major metabolites of Benzyl-4CN-BUTINACA, the second of which was a decyanation metabolite (B2). Both compounds also had a metabolite with *N*-dealkylation, meaning the loss of a pentanenitrile fragment. The pentanenitrile fragment could then lead to a loss of cyanide; however, since this was unable to be confirmed, the dealkylated metabolites are not reported as cyanide-releasing. In comparison, Åstrand et al. found 95.4% and 43.0% of Cumyl-4CN-BUTINACA metabolites from HHeps incubations and authentic urine samples, respectively, had a loss of cyanide (Åstrand et al. 2018). Staeheli et al. also found decyanation to be a major biotransformation for Cumyl-4CN-BUTINACA and Cumyl-4CN-B7AICA following incubation with HHeps (Staeheli et al. 2018). This demonstrates the amount of decyanation that occurs depends heavily on the head group of the SCRA. The unsubstituted aromatic and cycloalkane (e.g., cumyl and benzyl, respectively) head groups are resistant to metabolism, so more metabolites have biotransformations on the core and tail, where decyanation has been found to be the main biotransformation on the tail for SCRAs with an aliphatic nitrile. The metabolism of additional nitrile-containing SCRAs should be examined to further explore the impact of different structural moieties on the release of cyanide.

## Conclusion

Given that the metabolites with a dihydrodiol on the indazole core (B1) and decyanation to a carboxylic acid (B2) are by far the most abundant of the nine metabolites identified for Benzyl-4CN-BUTINACA following incubation with HHeps, these metabolites along with the parent drug are suggested as suitable urinary markers to identify consumption of Benzyl-4CN-BUTINACA. The metabolites with ester hydrolysis (M1) and ester hydrolysis combined with dehydrogenation (M2) were by far the most abundant of the 12 metabolites identified for MDMB-4CN-BUTINACA following incubation with HHeps: therefore, these metabolites are suggested as suitable urinary markers to identify consumption of MDMB-4CN-BUTINACA. It should be noted that these ester hydrolysis metabolites of MDMB-4CN-BUTINACA may also be formed from the metabolism of ADB-4CN-BUTINACA, although the metabolism of this SCRA have not yet been studied. In the future, the results of this study should be compared to the metabolic profile found in authentic case samples from the use of MDMB-4CN-BUTINACA and Benzyl-4CN-BUTINACA. In the meantime, it is recommended that clinical and forensic toxicologists add these metabolites and characteristic ions to their targeted and semi-targeted analytical methods. Although the amount of decyanation was not as prominent for Benzyl-4CN-BUTINACA and MDMB-4CN-BUTINACA as for Cumyl-4CN-BUTINACA, still 29 and 1.78%, respectively, of the metabolite peak area corresponded to decyanation.

## Supplementary Information

Below is the link to the electronic supplementary material.

Supplementary file1 (PPTX 661 KB)

Supplementary file2 (PPTX 1457 KB)

## Data Availability

All data supporting the findings of this study are available within the paper and its Supplementary Information.
