# Supplementary material for: In vitro metabolism of Benzyl-4CN-BUTINACA and MDMB-4CN-BUTINACA using human hepatocytes and LC-QToF-MS analysis
Source: Arch Toxicol. 2025 Mar 18;99(6):2355–66. doi: 10.1007/s00204-025-04018-y (PMC12185655; doi:10.1007/s00204-025-04018-y)
Supplement: Supplementary file 1 — Supplementary file1 (PPTX 661 KB) [file 204_2025_4018_MOESM1_ESM.pptx]

## Slide 1
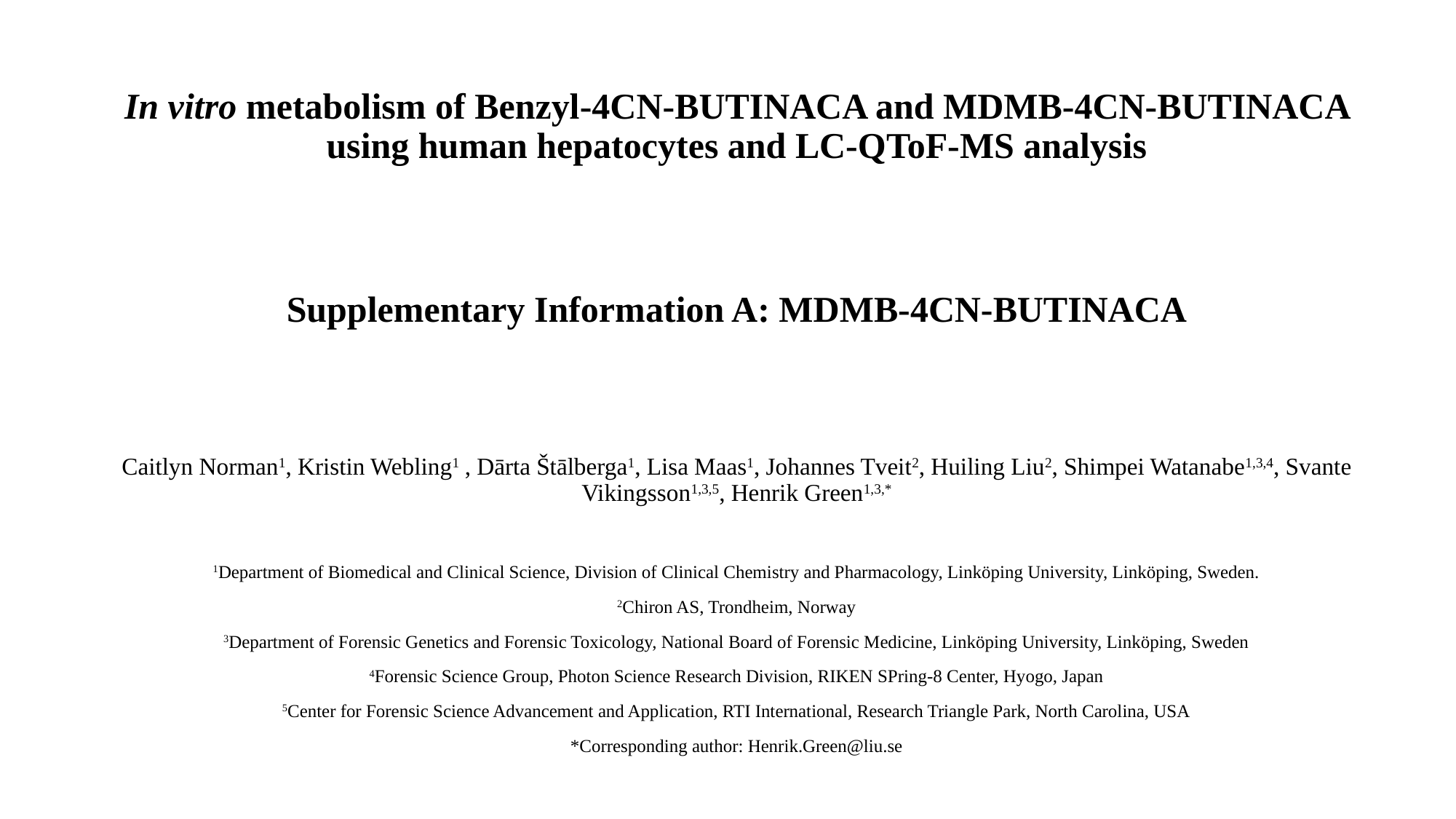

In vitro metabolism of Benzyl-4CN-BUTINACA and MDMB-4CN-BUTINACA using human hepatocytes and LC-QToF-MS analysis
Supplementary Information A: MDMB-4CN-BUTINACA
Caitlyn Norman1, Kristin Webling1 , Dārta Štālberga1, Lisa Maas1, Johannes Tveit2, Huiling Liu2, Shimpei Watanabe1,3,4, Svante Vikingsson1,3,5, Henrik Green1,3,*
1Department of Biomedical and Clinical Science, Division of Clinical Chemistry and Pharmacology, Linköping University, Linköping, Sweden.
2Chiron AS, Trondheim, Norway
3Department of Forensic Genetics and Forensic Toxicology, National Board of Forensic Medicine, Linköping University, Linköping, Sweden
4Forensic Science Group, Photon Science Research Division, RIKEN SPring-8 Center, Hyogo, Japan
5Center for Forensic Science Advancement and Application, RTI International, Research Triangle Park, North Carolina, USA
*Corresponding author: Henrik.Green@liu.se

## Slide 2
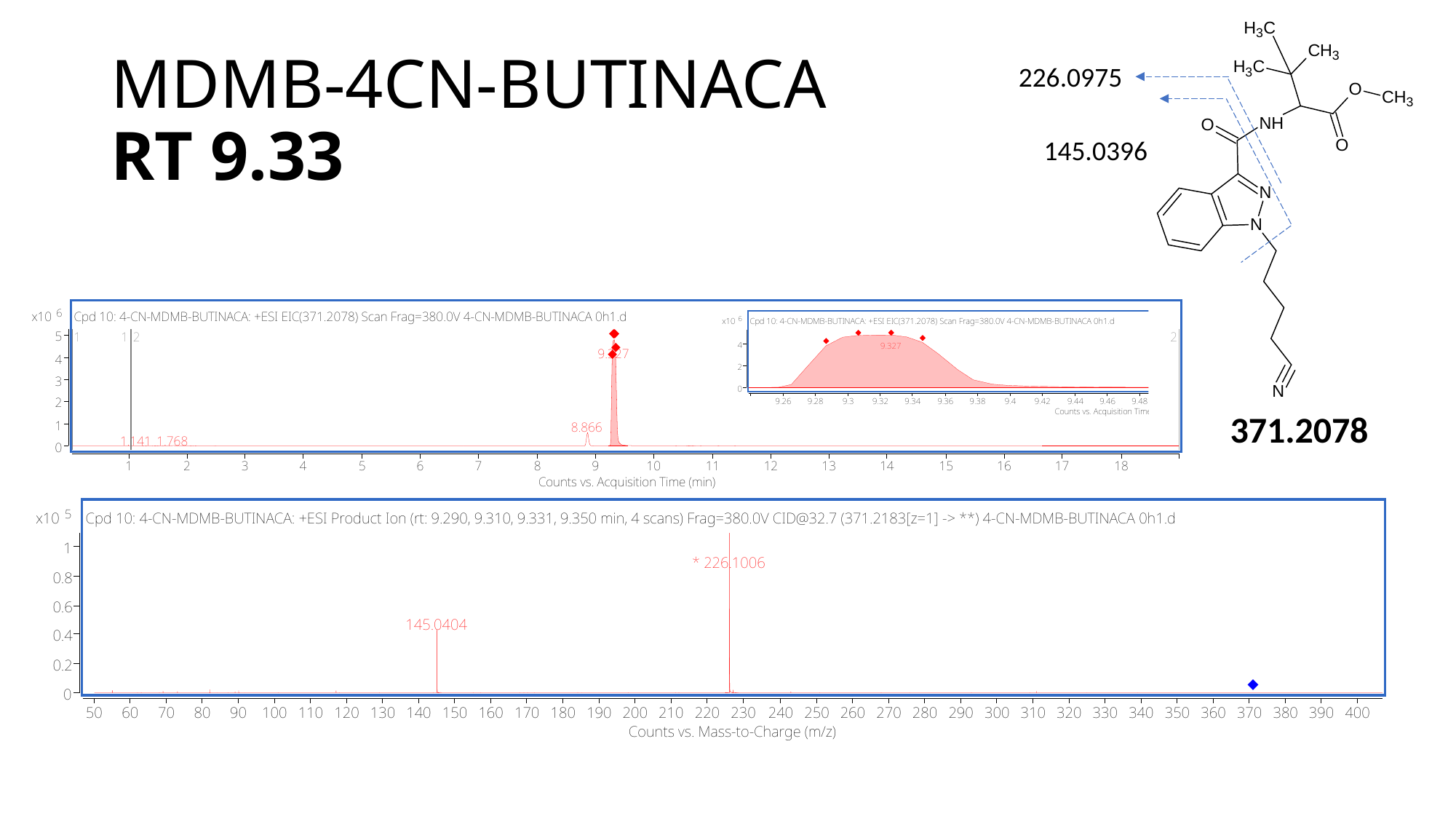

# MDMB-4CN-BUTINACA RT 9.33
226.0975
145.0396
371.2078

## Slide 3
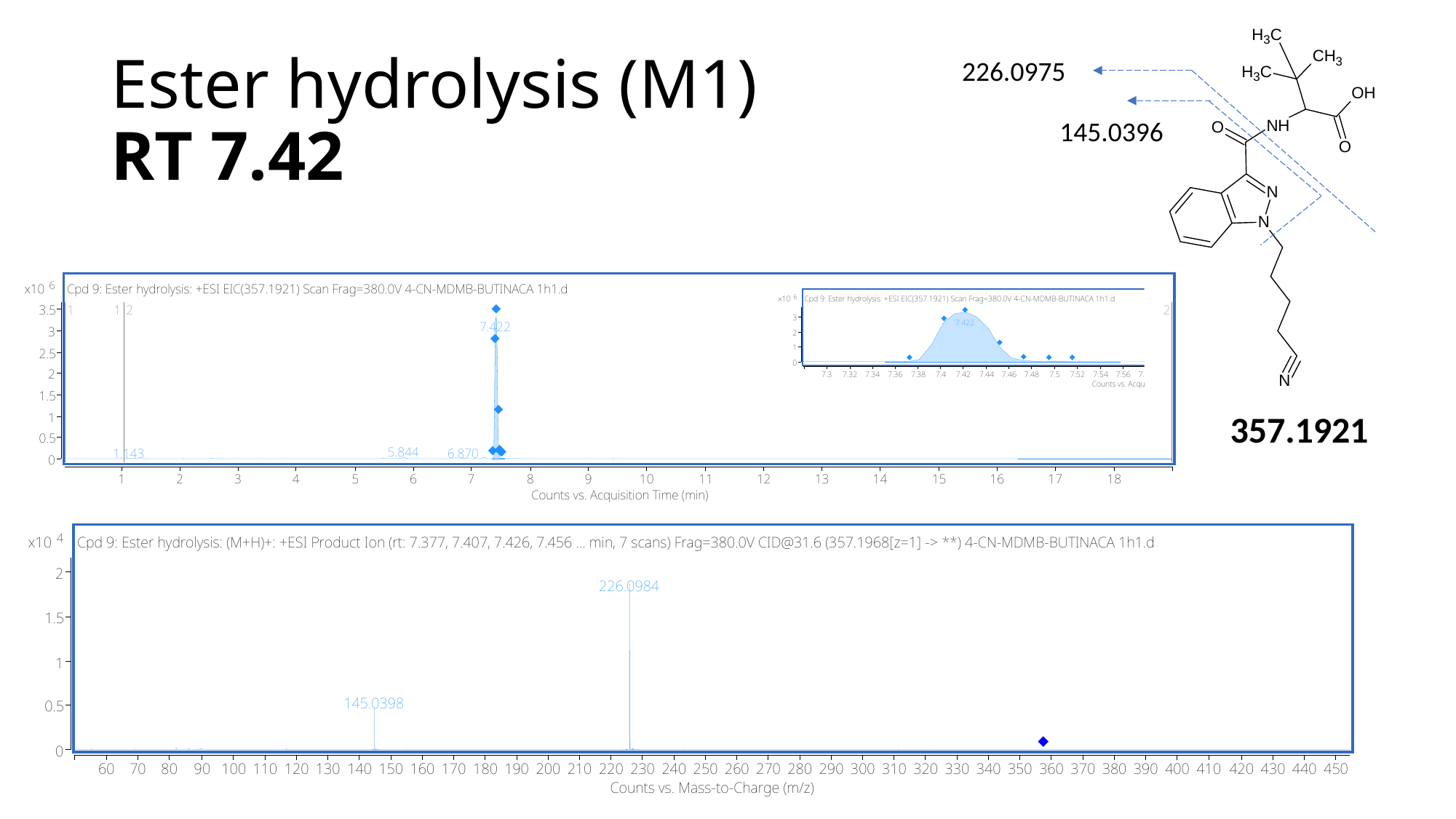

# Ester hydrolysis (M1) RT 7.42
226.0975
145.0396
357.1921

## Slide 4
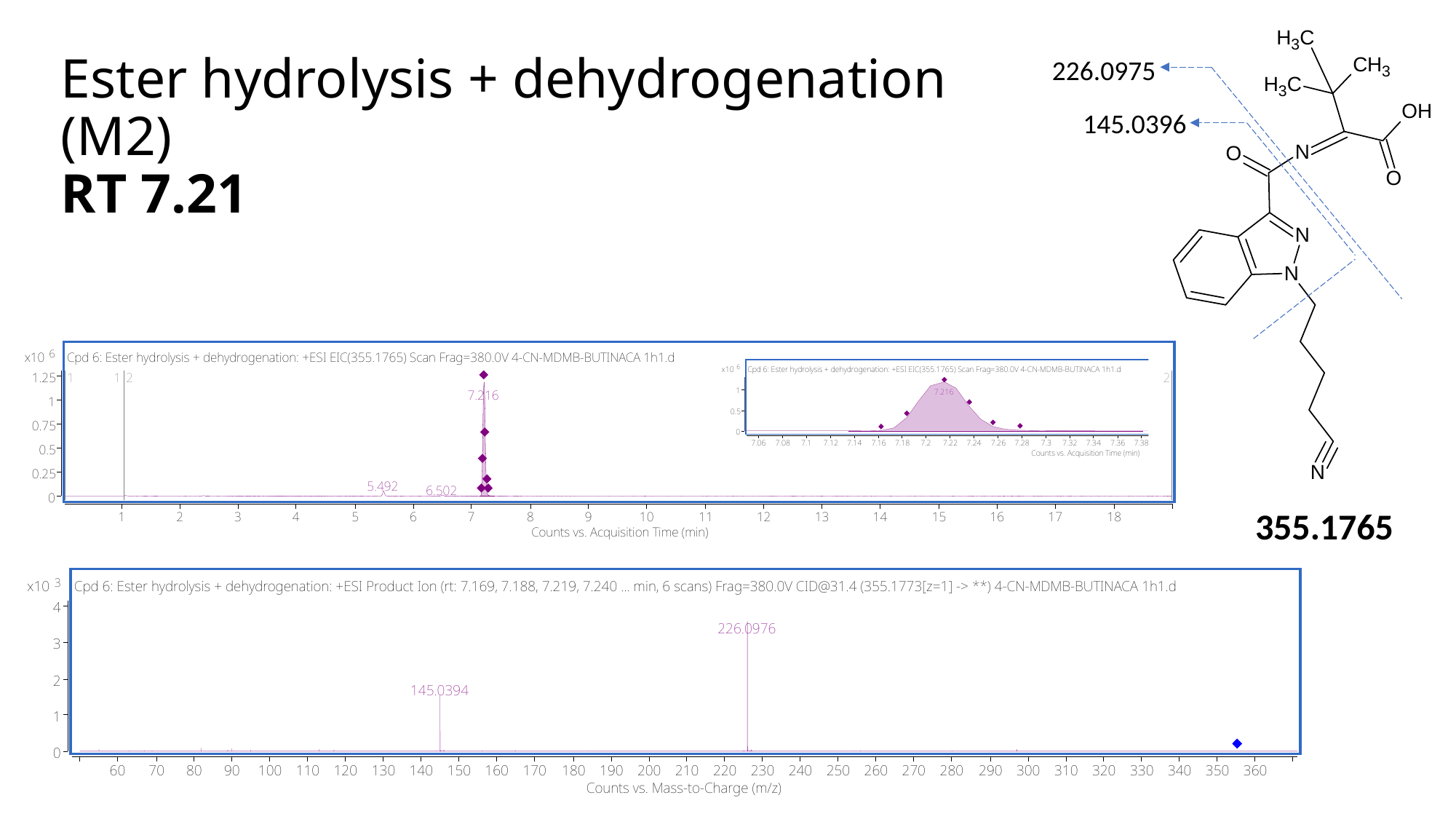

# Ester hydrolysis + dehydrogenation (M2) RT 7.21
226.0975
145.0396
355.1765

## Slide 5
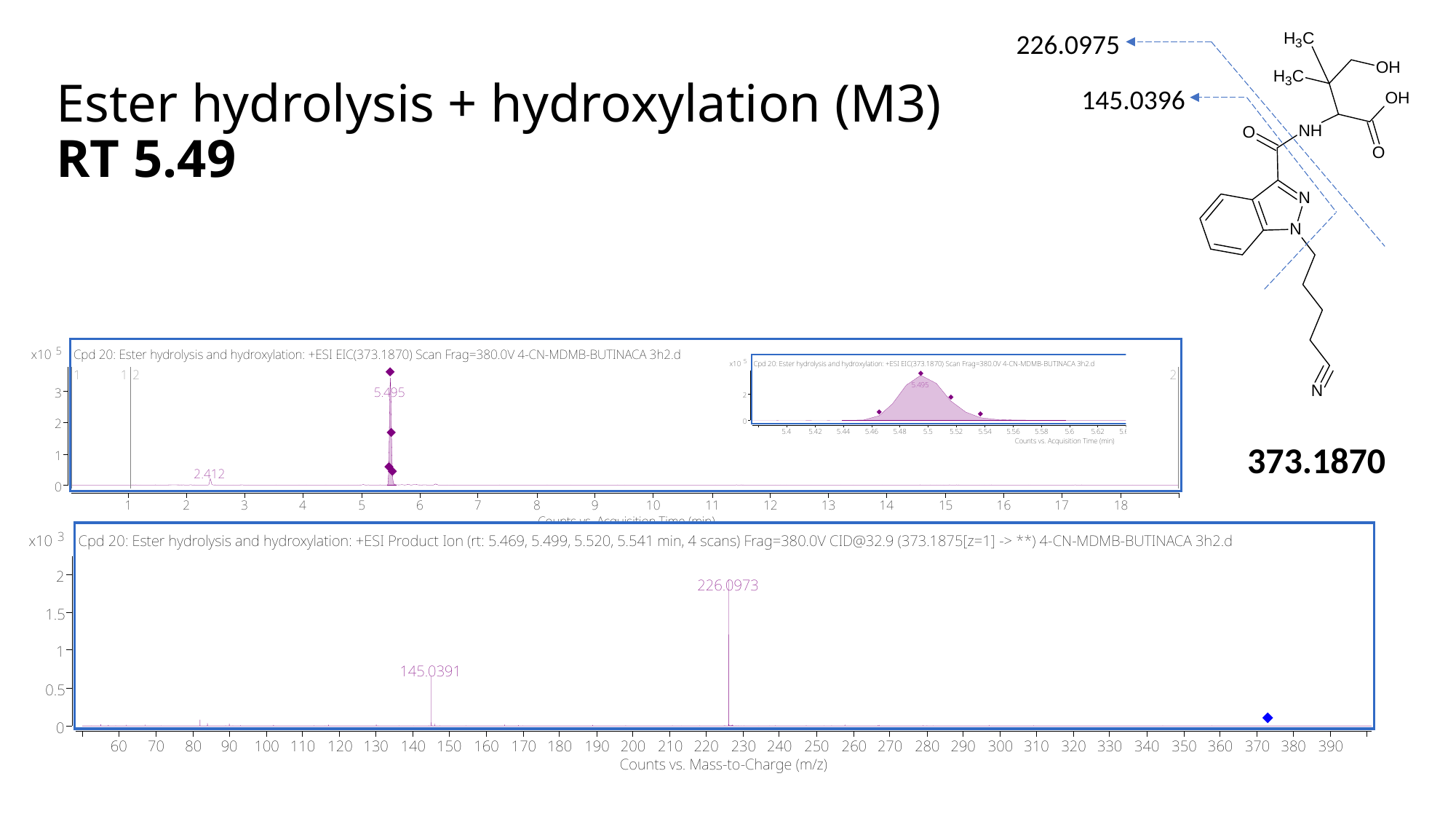

226.0975
# Ester hydrolysis + hydroxylation (M3) RT 5.49
145.0396
373.1870

## Slide 6
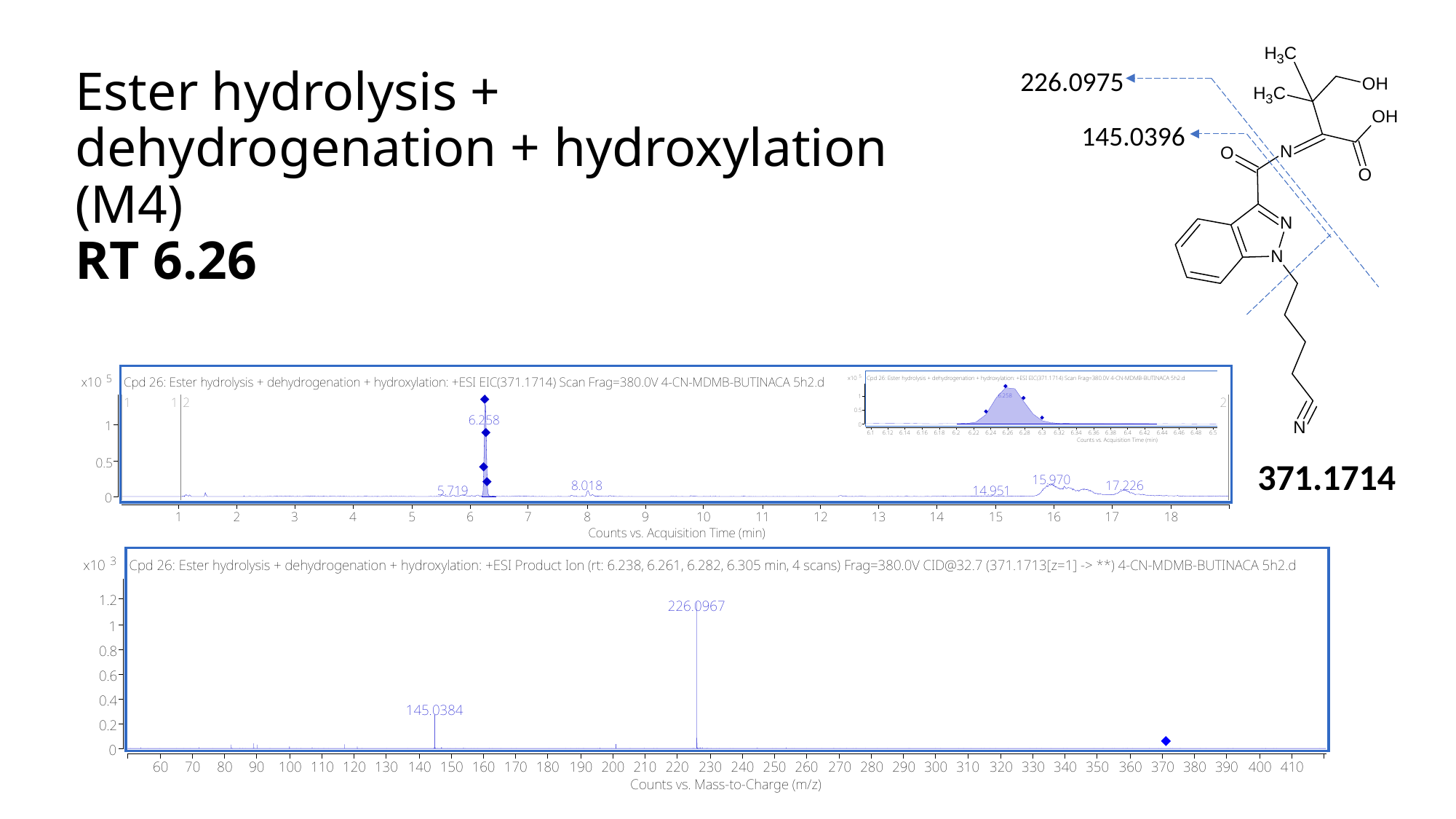

226.0975
# Ester hydrolysis + dehydrogenation + hydroxylation (M4) RT 6.26
145.0396
371.1714

## Slide 7
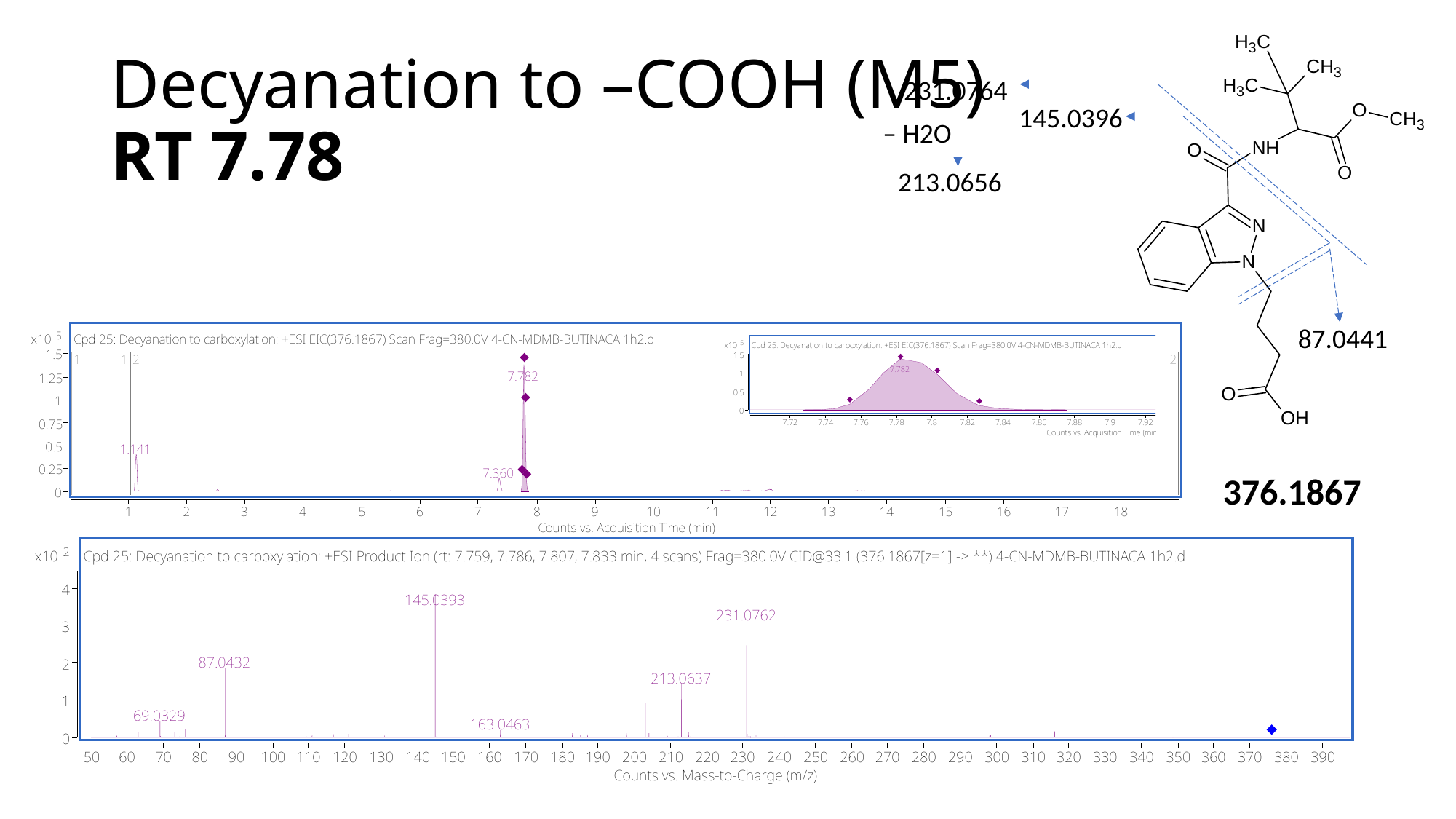

# Decyanation to –COOH (M5) RT 7.78
231.0764
145.0396
– H2O
213.0656
87.0441
376.1867

## Slide 8
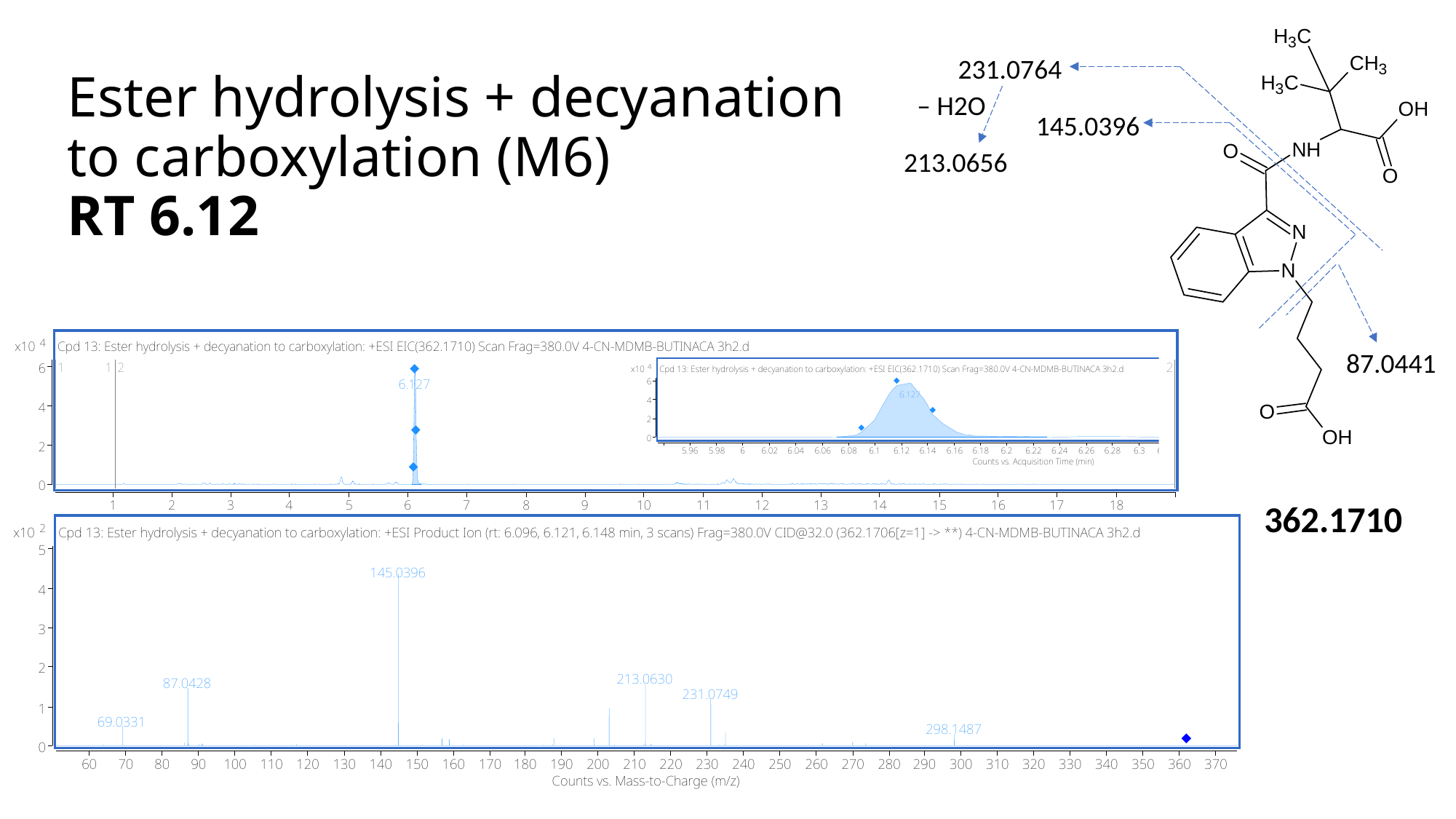

231.0764
# Ester hydrolysis + decyanation to carboxylation (M6) RT 6.12
– H2O
145.0396
213.0656
87.0441
362.1710

## Slide 9
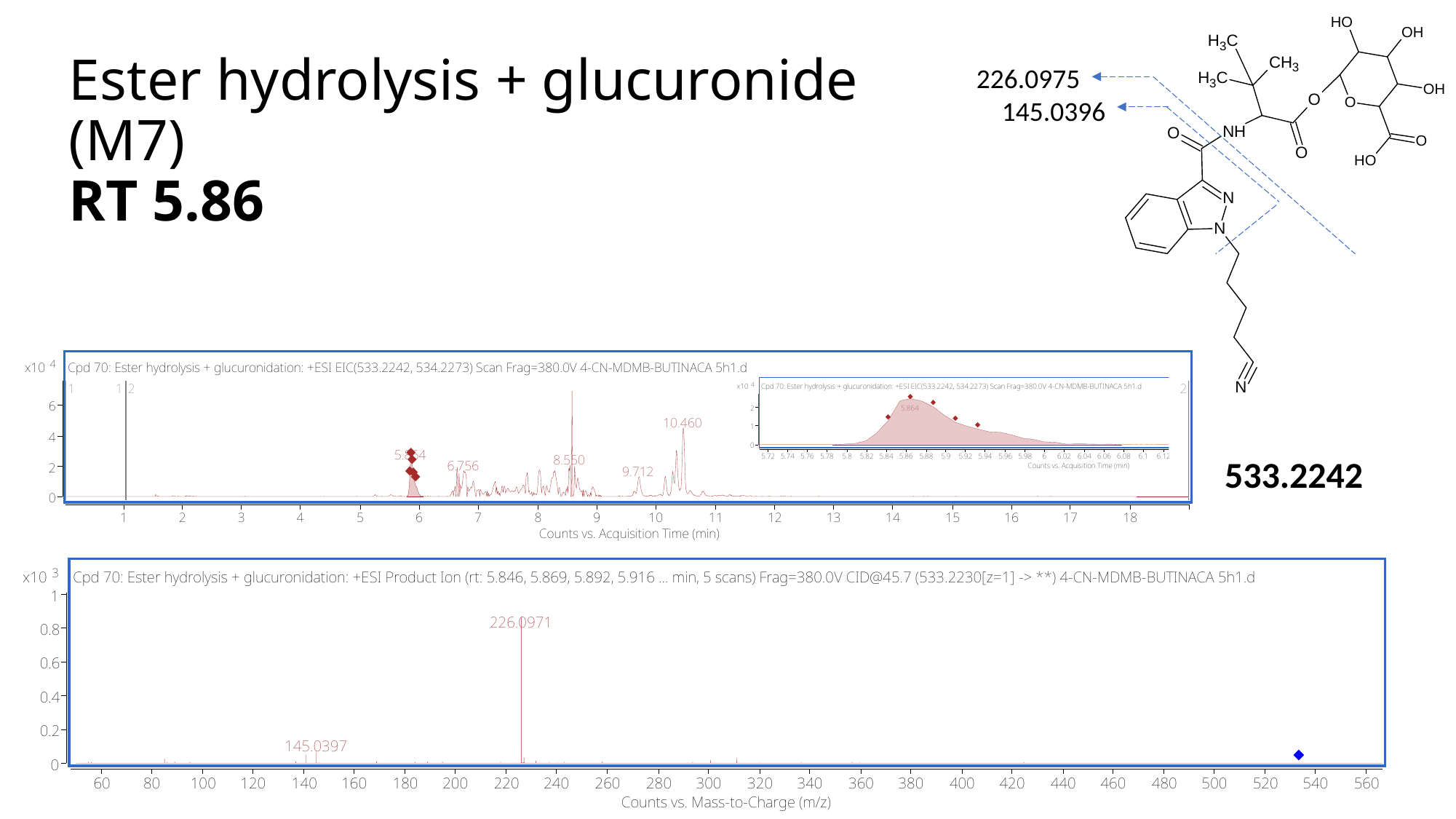

# Ester hydrolysis + glucuronide (M7) RT 5.86
226.0975
145.0396
533.2242

## Slide 10
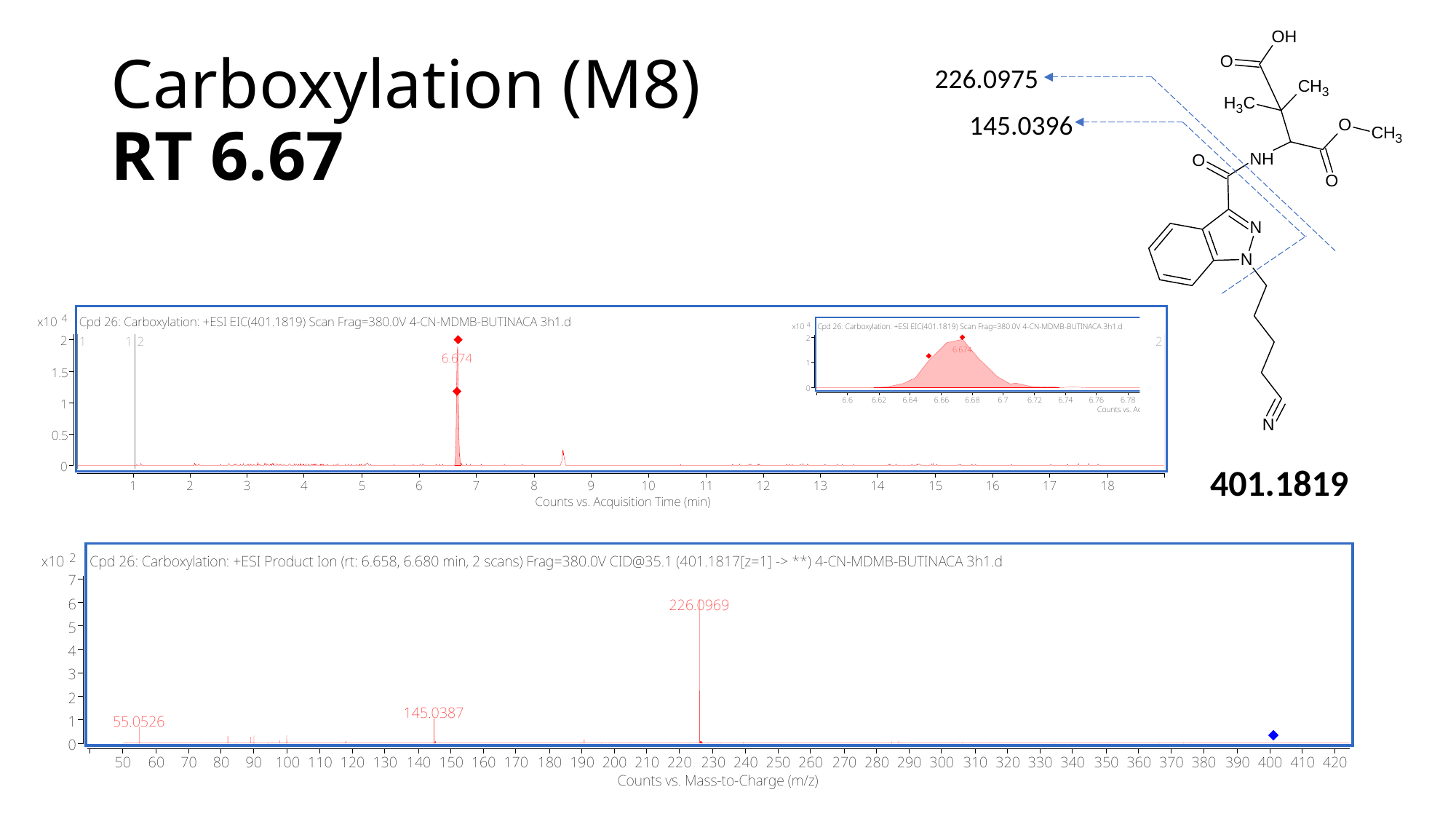

# Carboxylation (M8) RT 6.67
226.0975
145.0396
401.1819

## Slide 11
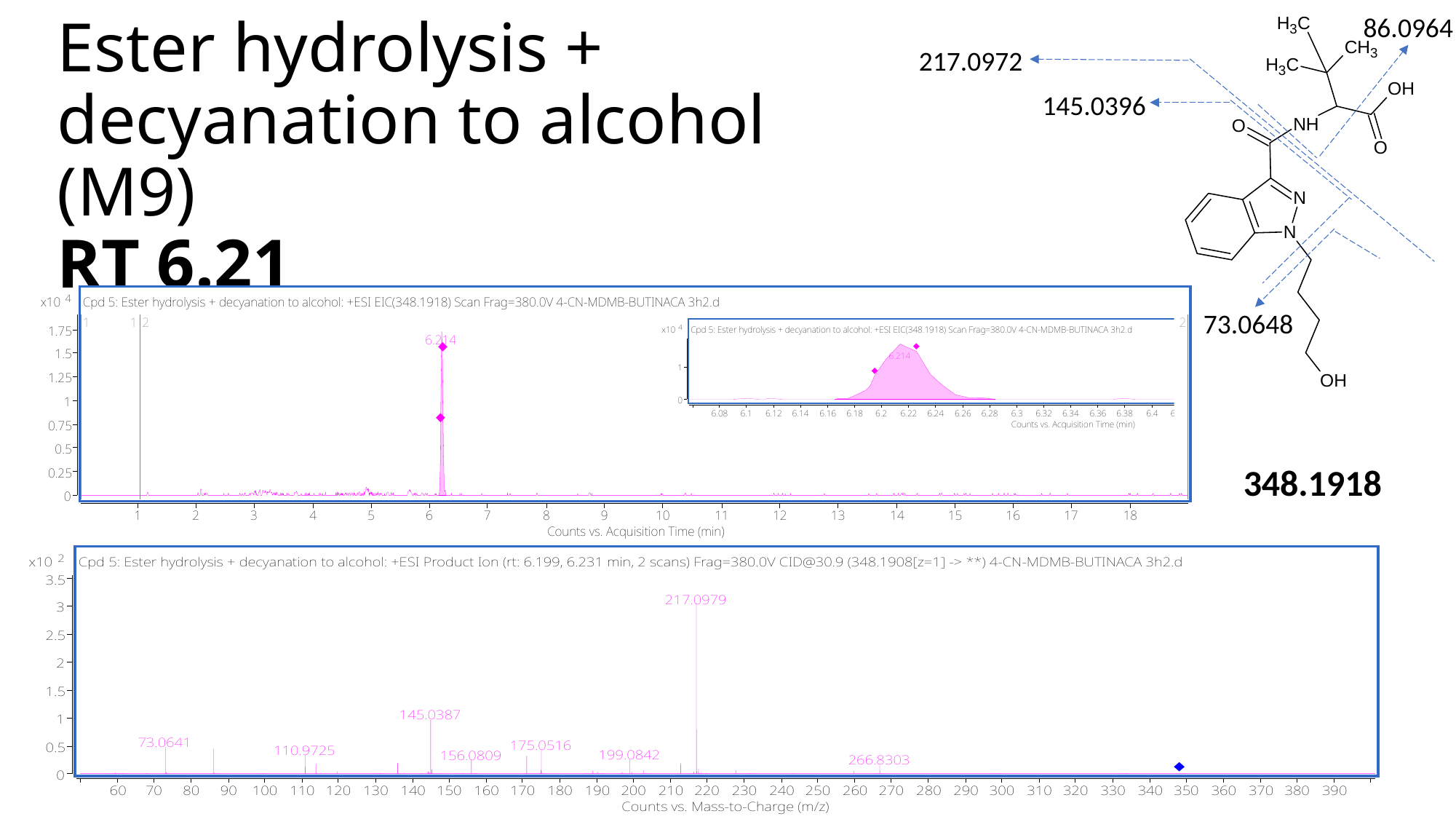

86.0964
# Ester hydrolysis + decyanation to alcohol (M9) RT 6.21
217.0972
145.0396
73.0648
348.1918

## Slide 12
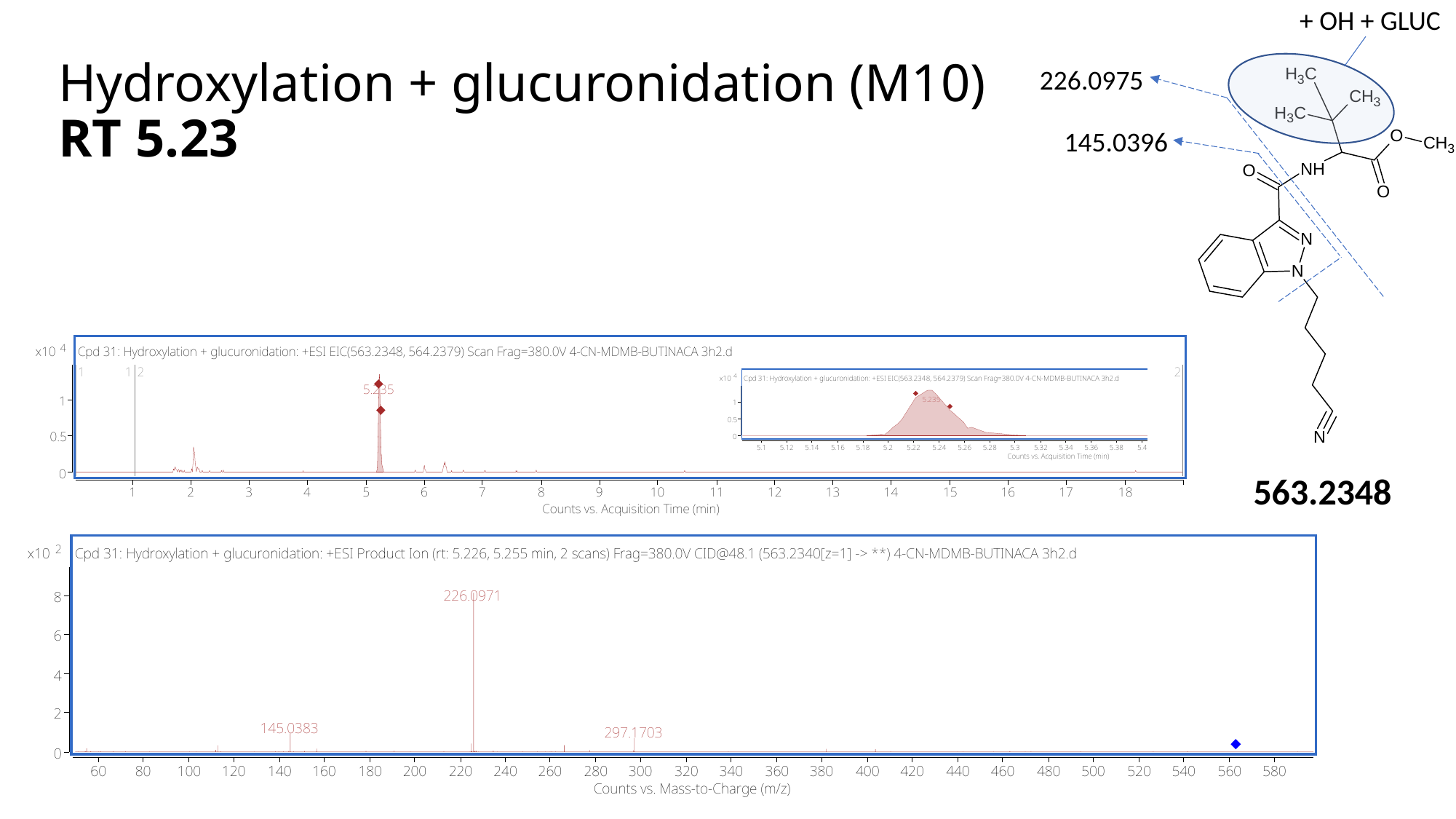

+ OH + GLUC
# Hydroxylation + glucuronidation (M10) RT 5.23
226.0975
145.0396
563.2348

## Slide 13
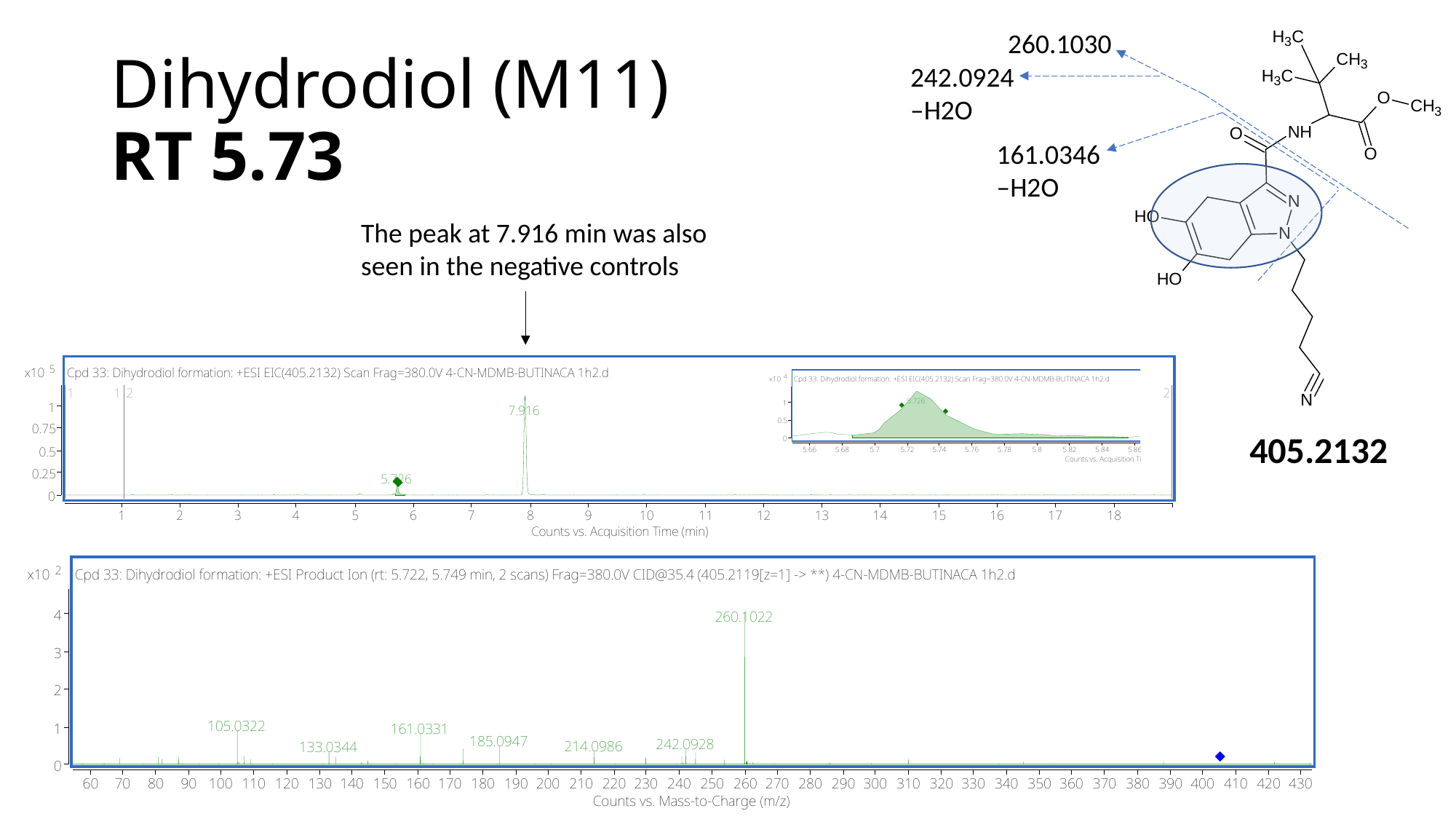

260.1030
# Dihydrodiol (M11) RT 5.73
242.0924
–H2O
161.0346 –H2O
The peak at 7.916 min was also seen in the negative controls
405.2132

## Slide 14
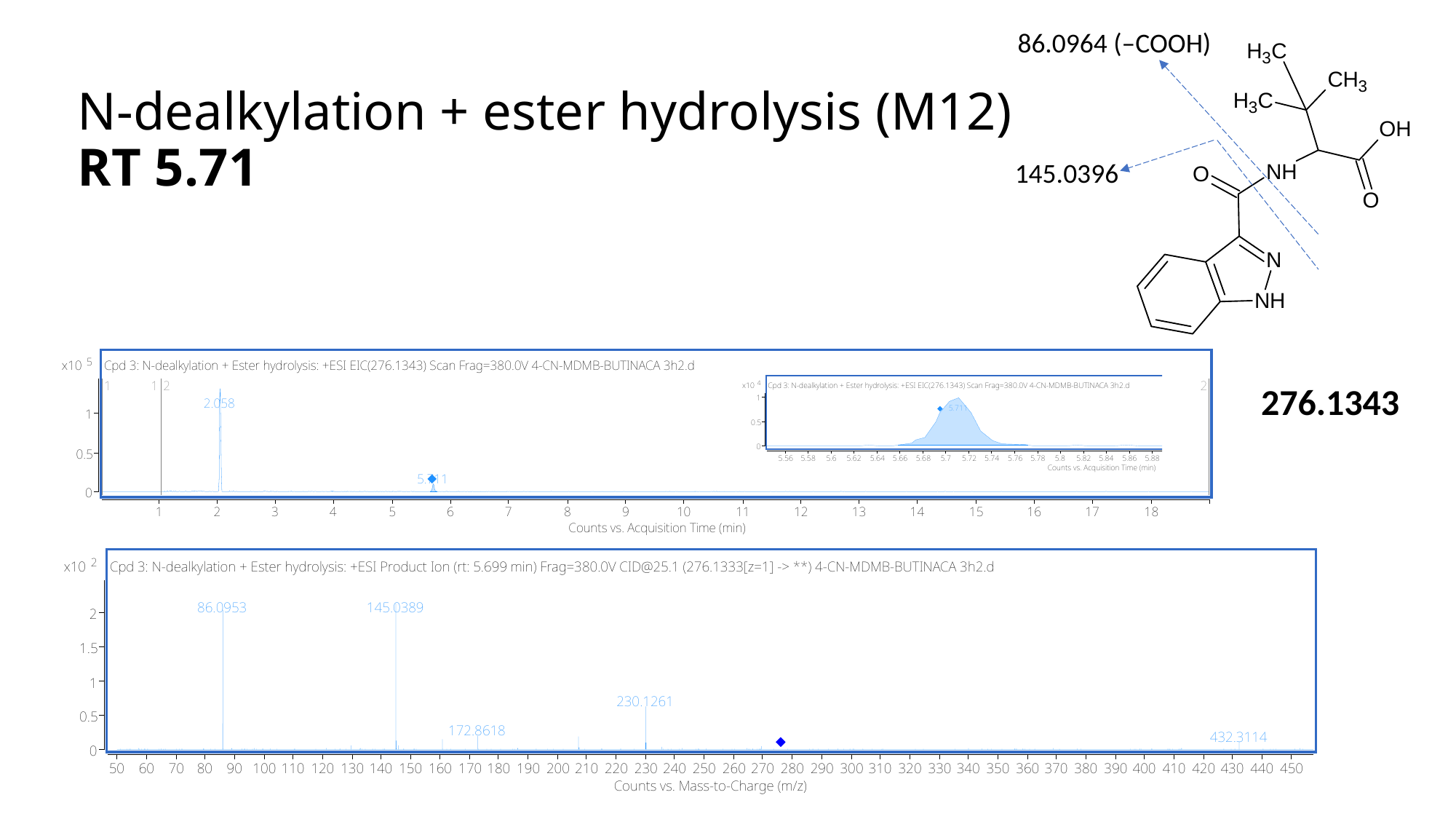

86.0964 (–COOH)
# N-dealkylation + ester hydrolysis (M12) RT 5.71
145.0396
276.1343
