# Supplementary material for: In vitro metabolism of Benzyl-4CN-BUTINACA and MDMB-4CN-BUTINACA using human hepatocytes and LC-QToF-MS analysis
Source: Arch Toxicol. 2025 Mar 18;99(6):2355–66. doi: 10.1007/s00204-025-04018-y (PMC12185655; doi:10.1007/s00204-025-04018-y)
Supplement: Supplementary file 2 — Supplementary file2 (PPTX 1457 KB) [file 204_2025_4018_MOESM2_ESM.pptx]

## Slide 1
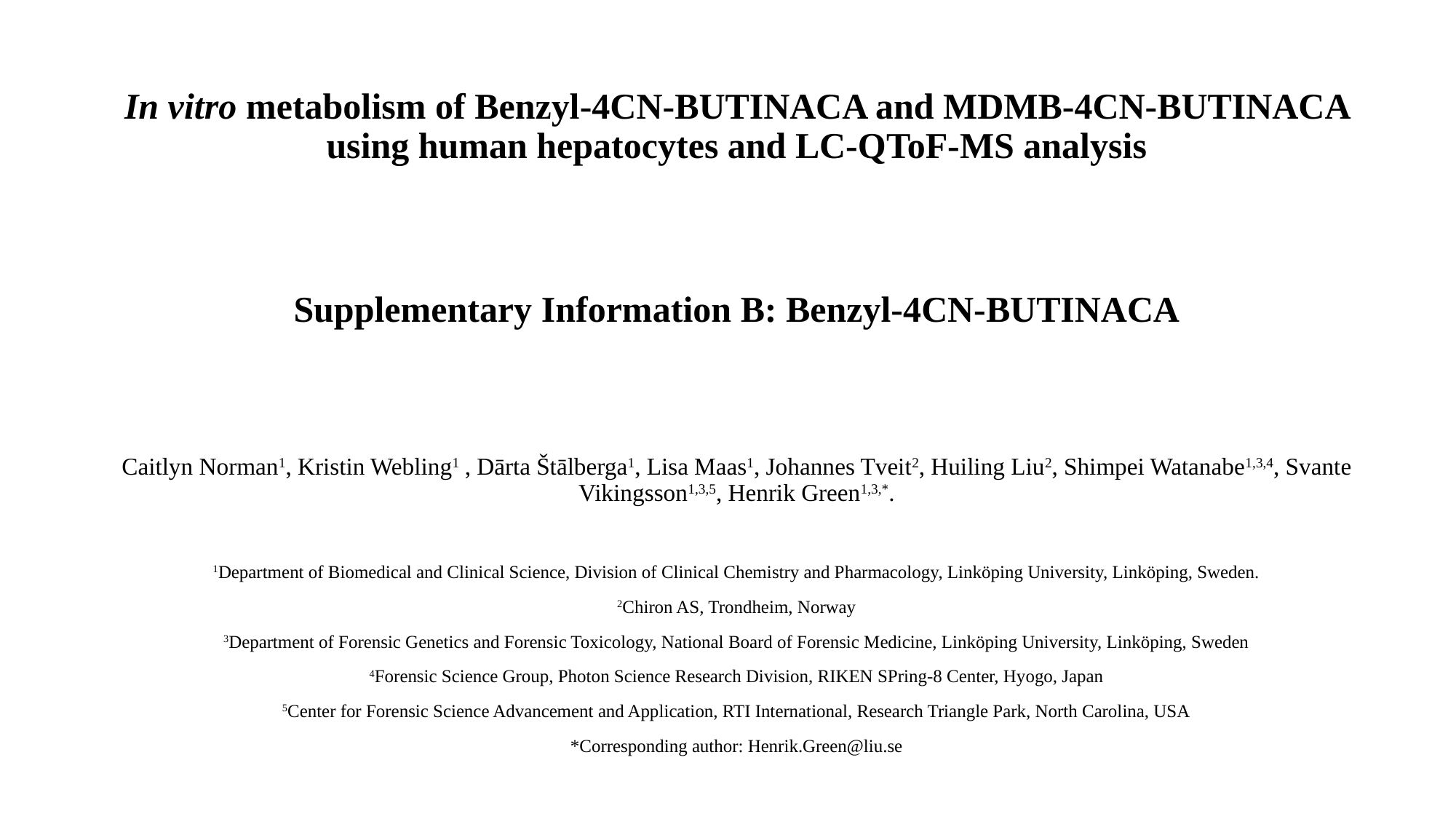

In vitro metabolism of Benzyl-4CN-BUTINACA and MDMB-4CN-BUTINACA using human hepatocytes and LC-QToF-MS analysis
Supplementary Information B: Benzyl-4CN-BUTINACA
Caitlyn Norman1, Kristin Webling1 , Dārta Štālberga1, Lisa Maas1, Johannes Tveit2, Huiling Liu2, Shimpei Watanabe1,3,4, Svante Vikingsson1,3,5, Henrik Green1,3,*.
1Department of Biomedical and Clinical Science, Division of Clinical Chemistry and Pharmacology, Linköping University, Linköping, Sweden.
2Chiron AS, Trondheim, Norway
3Department of Forensic Genetics and Forensic Toxicology, National Board of Forensic Medicine, Linköping University, Linköping, Sweden
4Forensic Science Group, Photon Science Research Division, RIKEN SPring-8 Center, Hyogo, Japan
5Center for Forensic Science Advancement and Application, RTI International, Research Triangle Park, North Carolina, USA
*Corresponding author: Henrik.Green@liu.se

## Slide 2
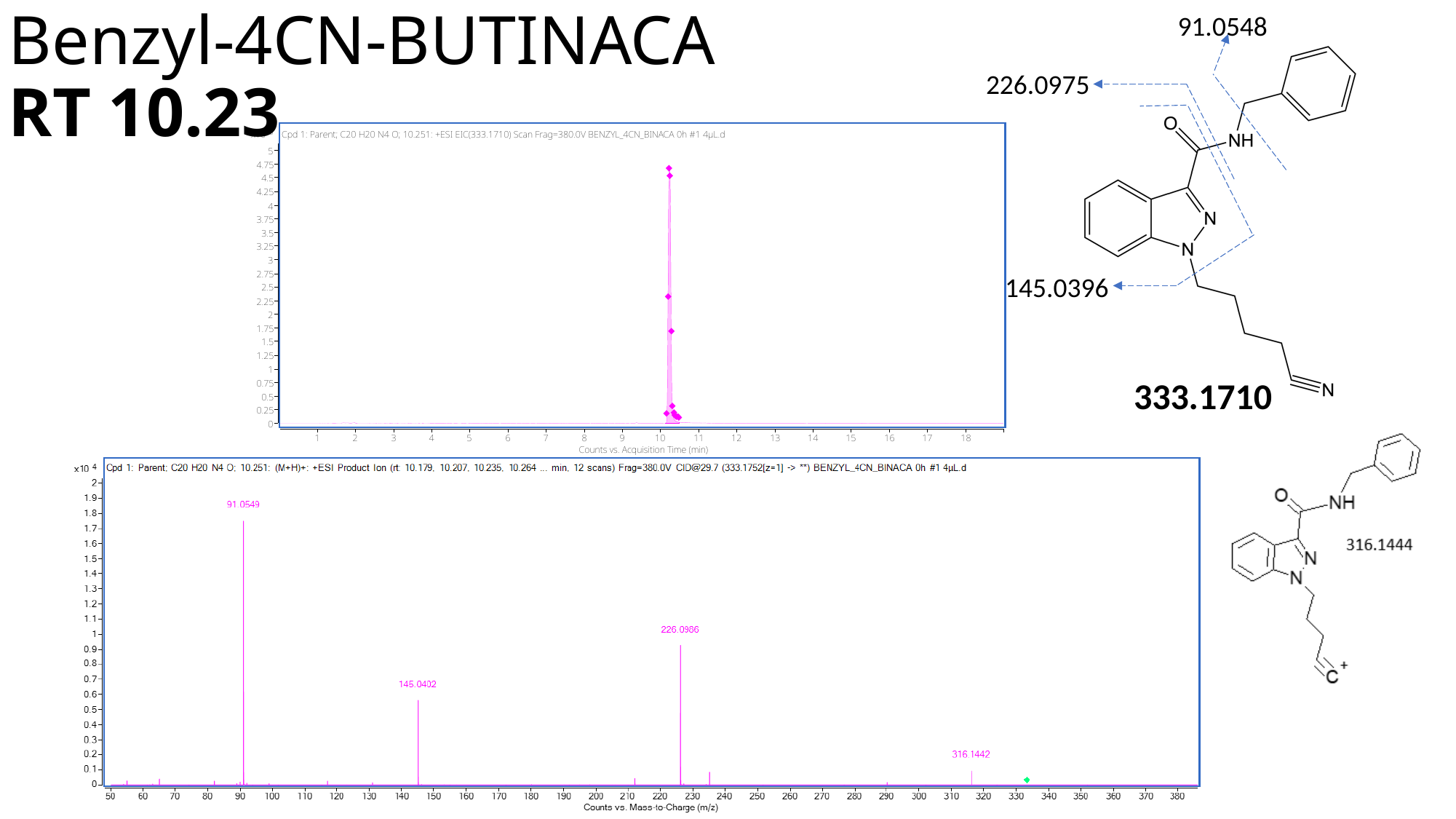

# Benzyl-4CN-BUTINACA RT 10.23
91.0548
226.0975
145.0396
333.1710

## Slide 3
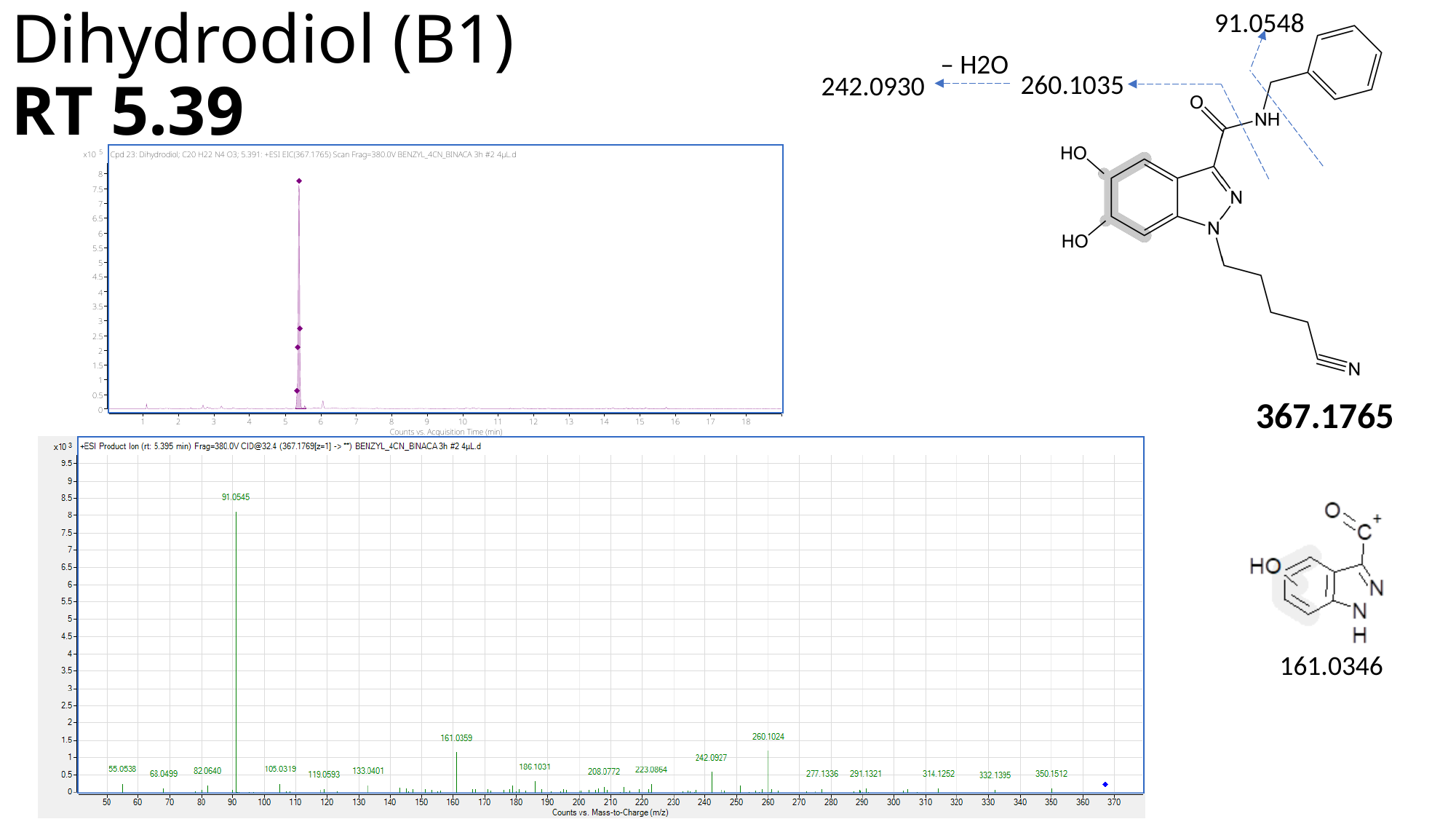

# Dihydrodiol (B1) RT 5.39
91.0548
– H2O
260.1035
242.0930
367.1765
161.0346

## Slide 4
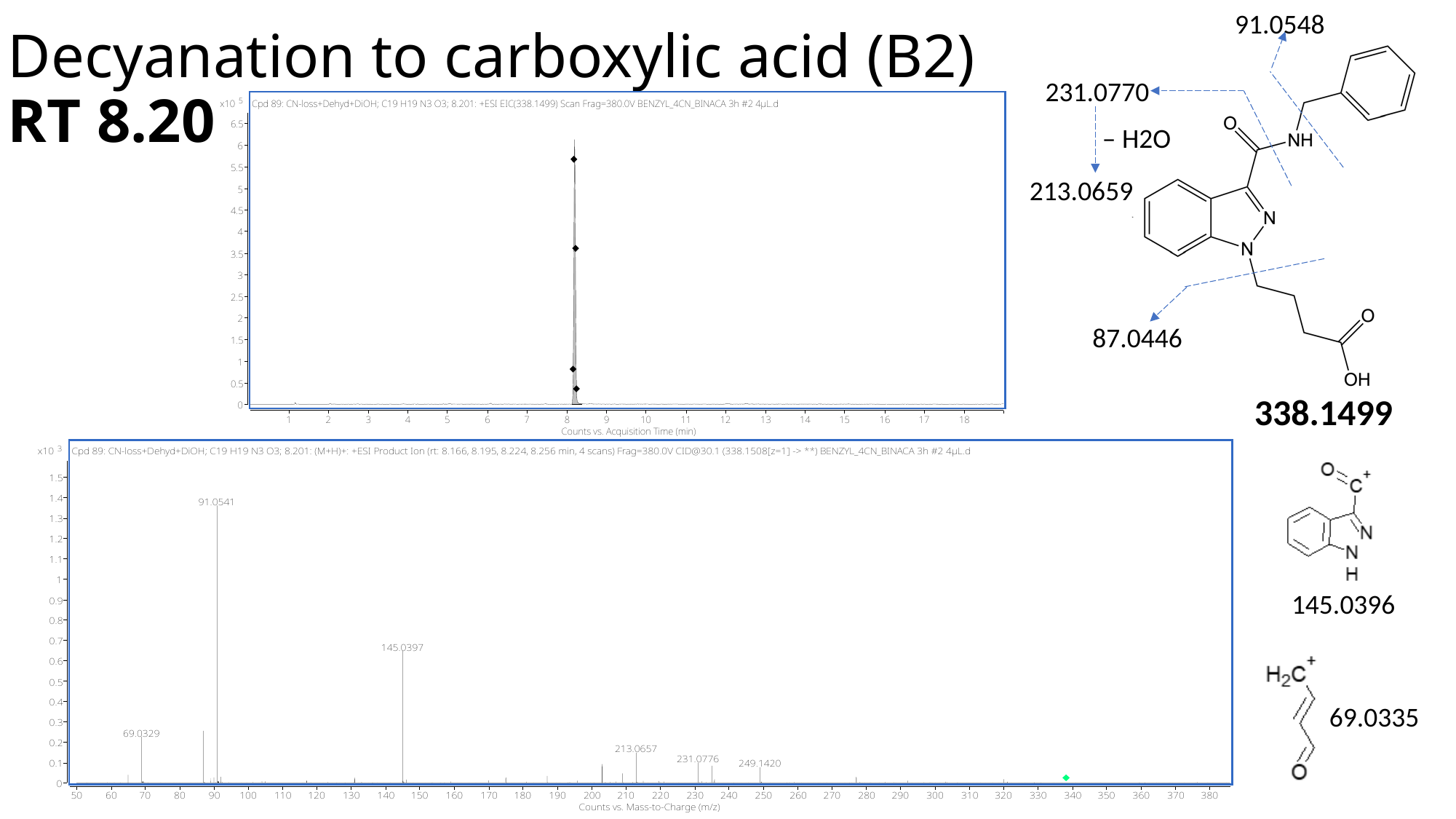

# Decyanation to carboxylic acid (B2) RT 8.20
91.0548
231.0770
– H2O
213.0659
87.0446
338.1499
145.0396
69.0335

## Slide 5
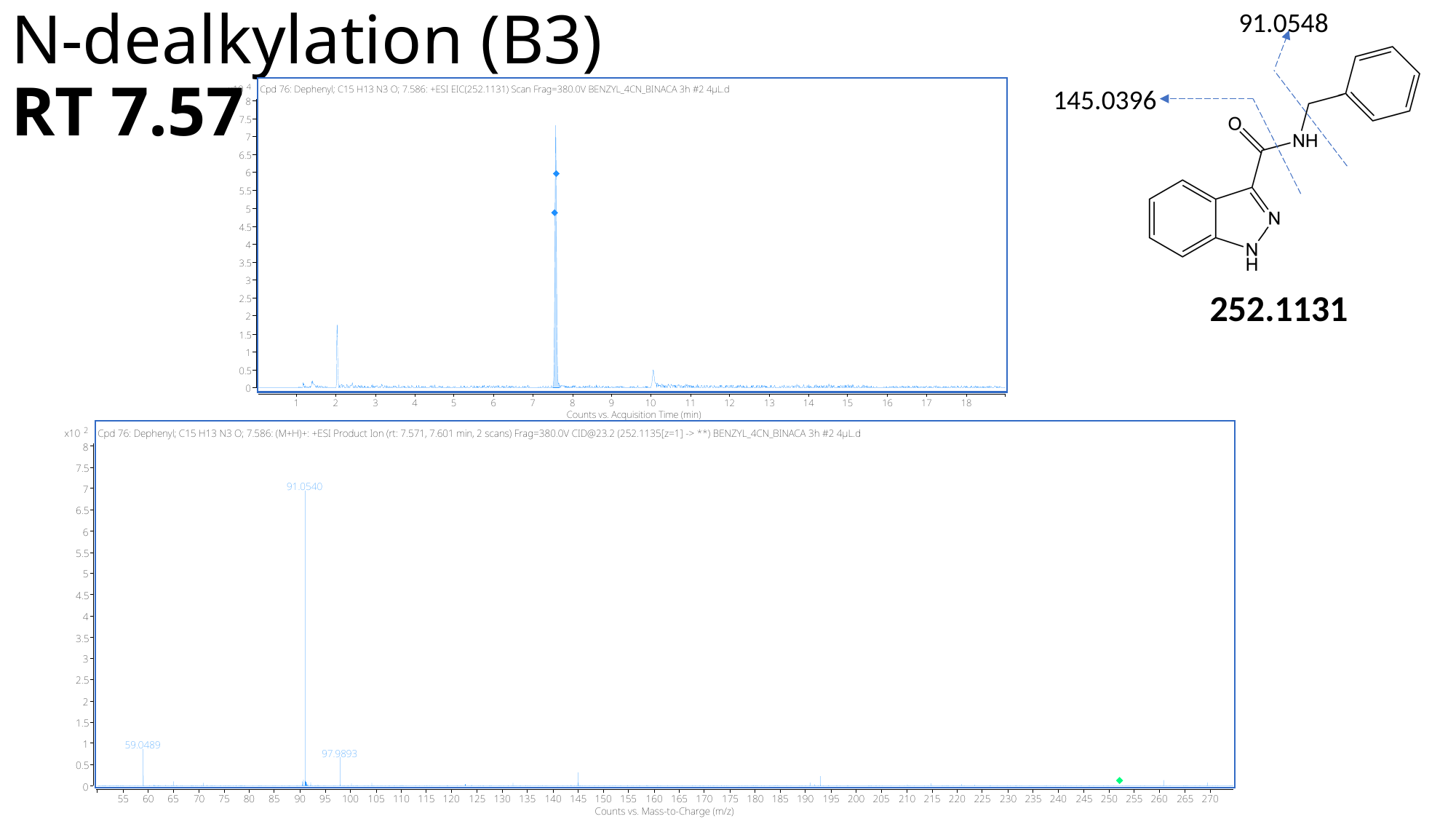

# N-dealkylation (B3) RT 7.57
91.0548
145.0396
252.1131

## Slide 6
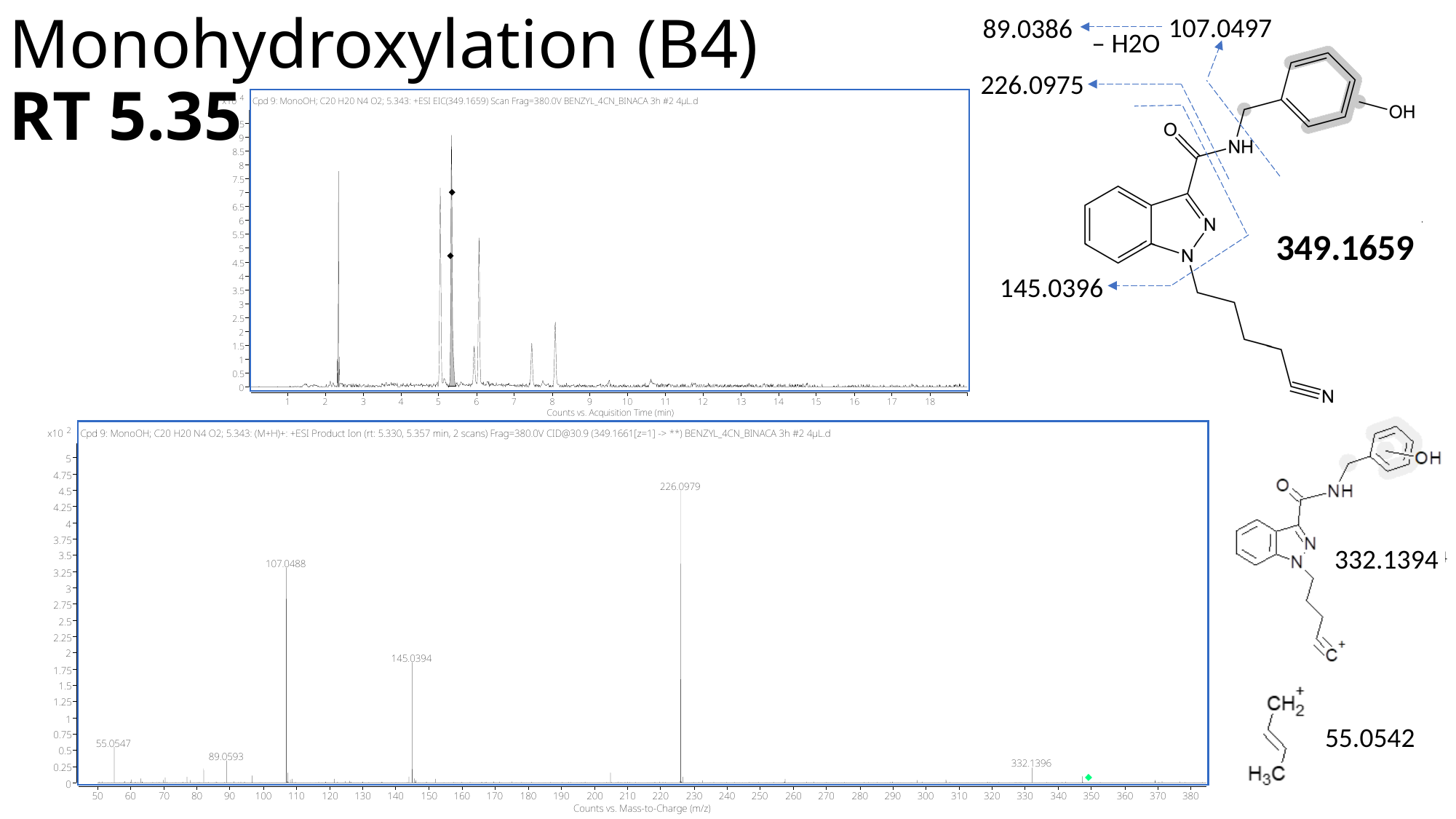

# Monohydroxylation (B4) RT 5.35
107.0497
89.0386
– H2O
226.0975
349.1659
145.0396
332.1394
55.0542

## Slide 7
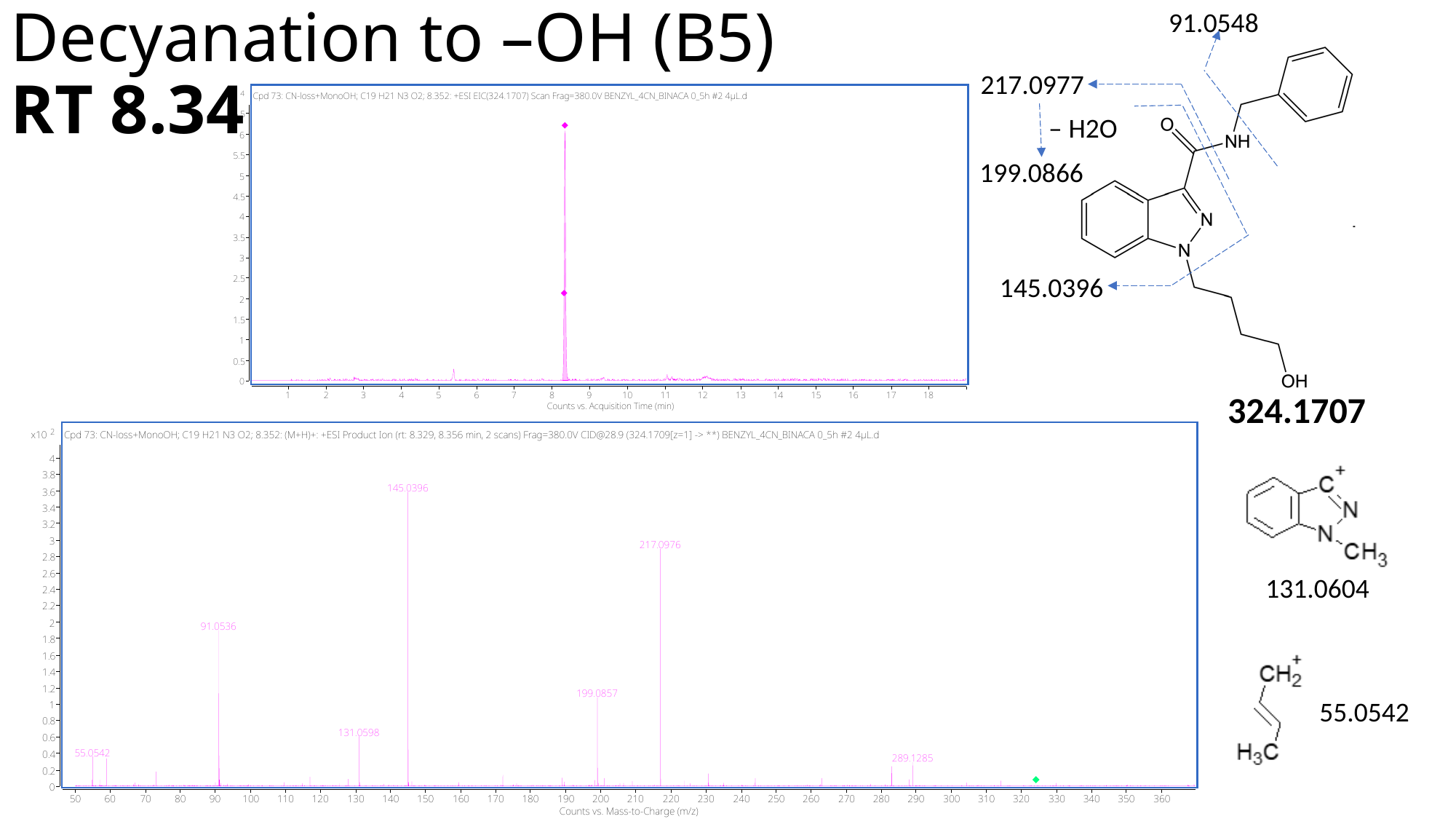

# Decyanation to –OH (B5) RT 8.34
91.0548
217.0977
– H2O
199.0866
145.0396
324.1707
131.0604
55.0542

## Slide 8
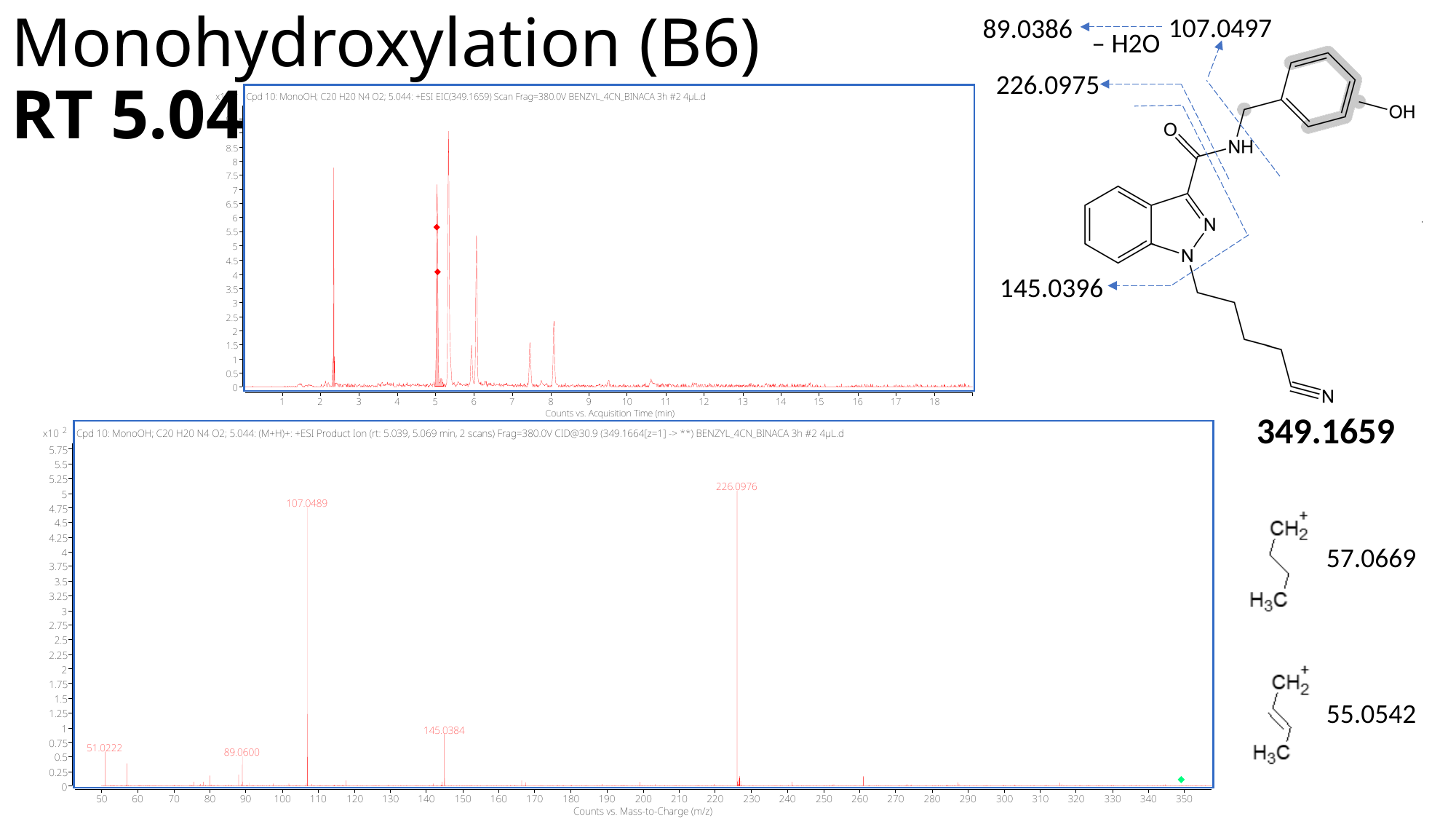

# Monohydroxylation (B6) RT 5.04
107.0497
89.0386
– H2O
226.0975
145.0396
349.1659
57.0669
55.0542

## Slide 9
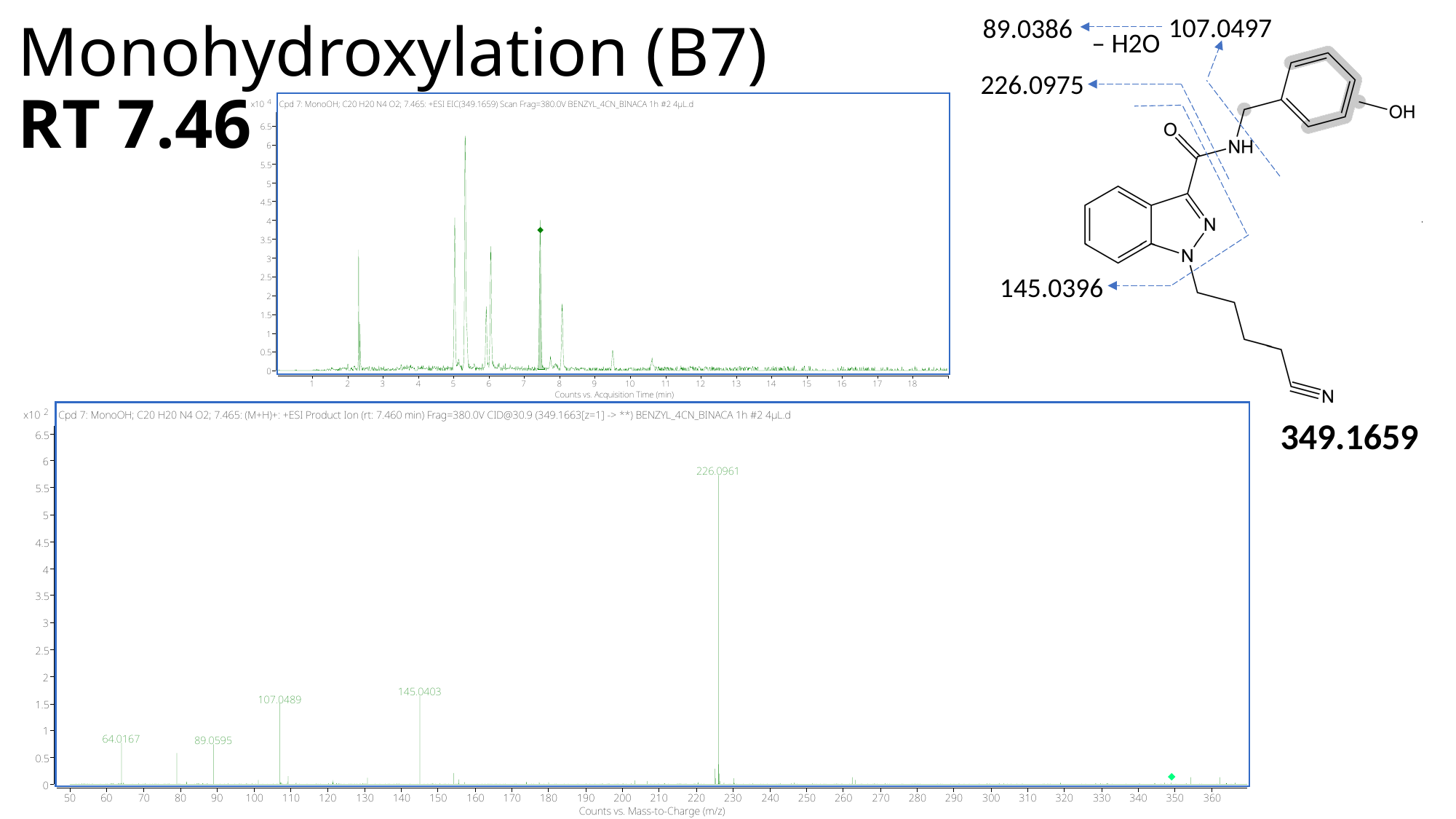

107.0497
89.0386
# Monohydroxylation (B7) RT 7.46
– H2O
226.0975
145.0396
349.1659

## Slide 10
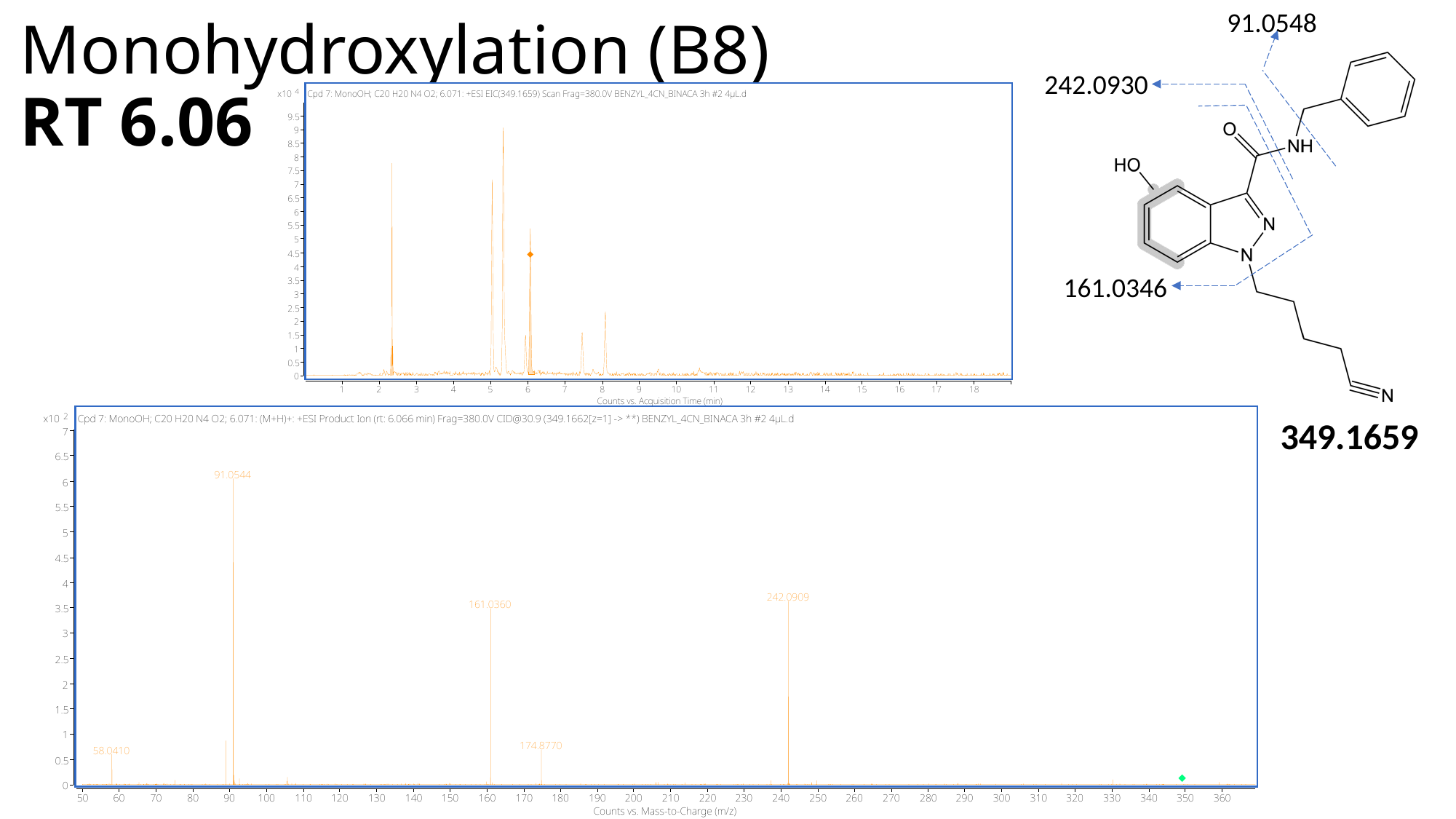

91.0548
# Monohydroxylation (B8) RT 6.06
242.0930
161.0346
349.1659

## Slide 11
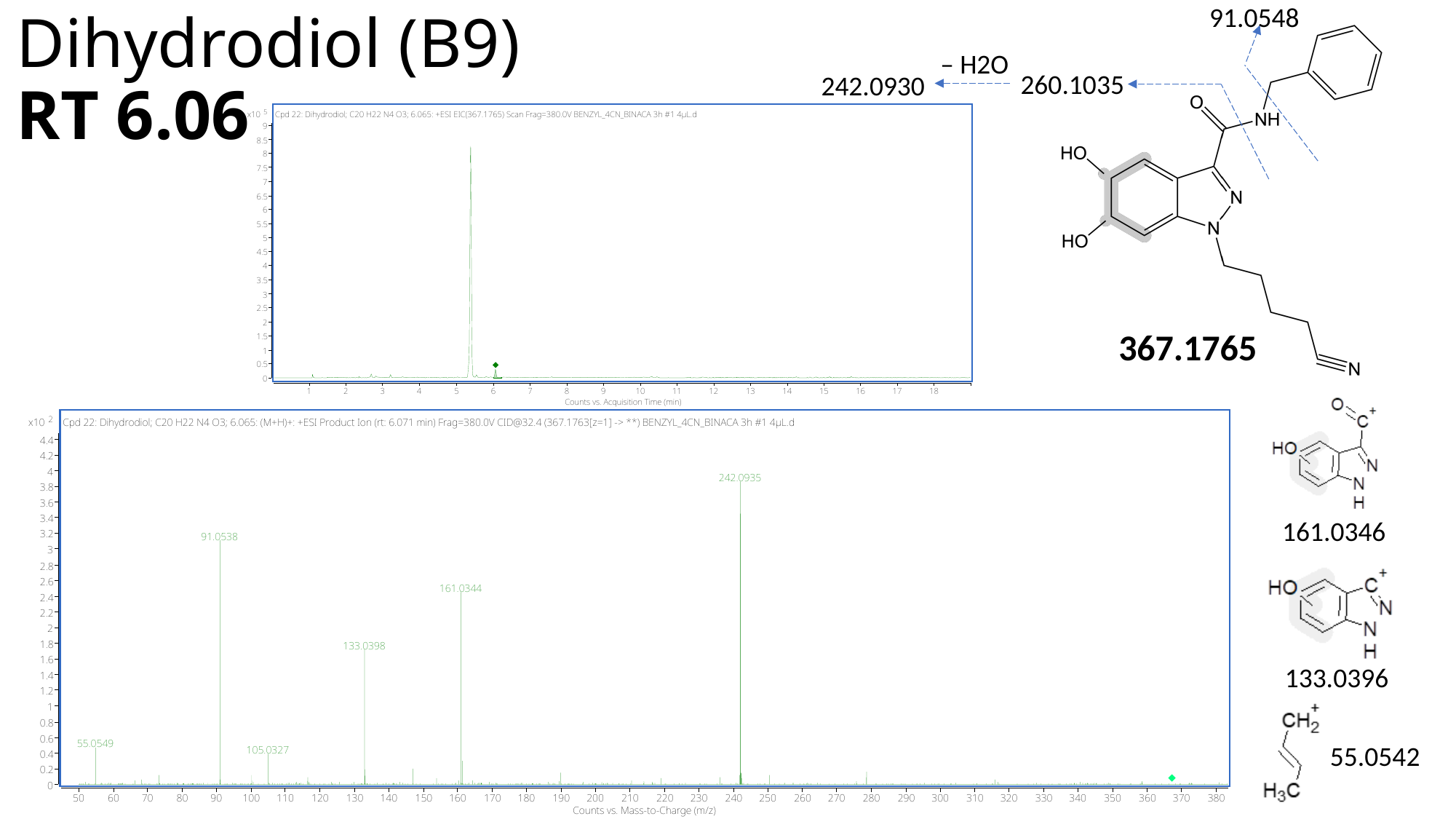

91.0548
# Dihydrodiol (B9) RT 6.06
– H2O
260.1035
242.0930
367.1765
161.0346
133.0396
55.0542
